# Multinuclear metal-binding ability of a carotene

**DOI:** 10.1038/ncomms7742

**Published:** 2015-04-10

**Authors:** Shinnosuke Horiuchi, Yuki Tachibana, Mitsuki Yamashita, Koji Yamamoto, Kohei Masai, Kohei Takase, Teruo Matsutani, Shiori Kawamata, Yuki Kurashige, Takeshi Yanai, Tetsuro Murahashi

**Affiliations:** 1 Research Center of Integrative Molecular Systems (CIMoS), Institute for Molecular Science, National Institutes of Natural Sciences, Myodaiji, Okazaki, Aichi 444-8787, Japan; 2 Department of Applied Chemistry, Graduate School of Engineering, Osaka University, Suita, Osaka 565-0871, Japan; 3 Department of Structural Molecular Science, The Graduate University for Advanced Studies, Myodaiji, Okazaki, Aichi 444-8787, Japan; 4 Department of Theoretical and Computational Molecular Science, Institute for Molecular Science, National Institutes of Natural Sciences, Myodaiji, Okazaki, Aichi 444-8585, Japan; 5 Department of Functional Molecular Science, The Graduate University for Advanced Studies, Myodaiji, Okazaki, Aichi 444-8585, Japan; 6 PRESTO, Japan Science and Technology Agency (JST), Myodaiji, Okazaki, Aichi 444-8787, Japan

## Abstract

Carotenes are naturally abundant unsaturated hydrocarbon pigments, and their fascinating physical and chemical properties have been studied intensively not only for better understanding of the roles in biological processes but also for the use in artificial chemical systems. However, their metal-binding ability has been virtually unexplored. Here we report that β-carotene has the ability to assemble and align ten metal atoms to afford decanuclear homo- and heterometal chain complexes. The metallo–carotenoid framework shows reversible metalation–demetalation reactivity with multiple metals, which allows us to control the size of metal chains as well as the heterobimetallic composition and arrangement of the carotene-supported metal chains.

## Abstract (web summary)

Carotenes are naturally abundant, widely studied unsaturated hydrocarbon pigments but their metal-binding ability has been virtually unexplored. Here, the authors demonstrate that they can be used to reversibly assemble and align homo- and hetero-metallic decanuclear chain complexes.

## Graphical abstract

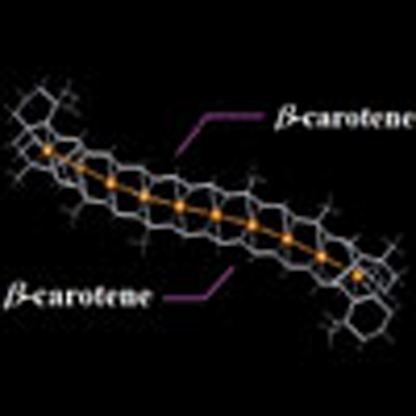

Carotenoids are naturally abundant pigments containing extended π-conjugated C=C double-bond arrays. The fascinating physical and chemical properties of carotenoids, such as light-harvesting, photo-protective, antioxidative and conductive properties have been explored not only for better understanding of their roles in biological processes but also for the use in artificial chemical systems^1^,^2^,^3^,^4^. An attractive, although yet undeveloped function of carotenoids is their metal-binding ability. While several early reports showed that carotenoids bind mononuclear metal moieties through conventional 1,3-butadiene-type tetrahapto–π-coordination^5^,^6^,^7^,^8^,^9^, the extended π-conjugated polyene moieties in their backbones may impart the ability to bind a large metal–metal (M-M)-bonded array through continuous multiple π-coordination bonds (Fig. 1). Such potential multidentate bridging π-coordination ability of carotenoids is highly attractive because of its usefulness in inorganic synthesis. Indeed, chemists have sought to use polynucleating multidentate ligands as the scaffolds or templates to control the metal assembly that provides methodology to synthesize molecularly well-defined metal clusters for catalysis and materials science^10^,^11^,^12^,^13^,^14^. However, the scaffold strategy has been applied mostly to the construction of metal clusters with small size owing to the difficulty to design practically useful large scaffold ligands that can assemble and then hold many metal atoms. In fact, only few synthetic scaffold ligands that can bind 10 or more metal atoms through multidentate bridging coordination have been developed. Recently, Peng and colleagues^15^ developed synthetic multidentate N-donor ligands that lead to isolation of Ni_11_ chain complexes. Shionoya and co-workers developed artificial metallo–DNA motifs that brought a method to construct a heterometal array of 10 metal ions, although direct M–M bonds are absent in the metal array^16^. While these previous studies used the σ-type scaffolds bearing multiple hetero-atom σ-binding sites, π-type extended π-conjugated unsaturated hydrocarbon scaffolds are also promising in light of their preferable rows of C=C π-binding sites at regular intervals comparable to M–M bond lengths^17^,^18^,^19^,^20^. Furthermore, relatively weak C=C π-coordination bond may cause dynamic metal binding at each C=C site, which would be a key for assembling many metal atoms in a convergent manner. Carotenes are the rational choice as the extended π-conjugated scaffold for many metal atoms, because carotenes are one of the most readily available extended unsaturated hydrocarbons containing more than ten π-conjugated C=C double bonds.

Herein, we report remarkable multinuclear metal-binding ability of β-carotene through synthesis and characterization of bis-(β-carotene) decanuclear metal chain complexes. The metallo–carotene framework shows interesting multinuclear metalation–demetalation reactivity, allowing us to construct heterobimetallic decanuclear chain. The bis-(β-carotene) decanuclear metal chain complexes are stable rod-like sandwich molecules, exhibiting parallel π–π stacking self-assembly in the crystalline state.

## Results

### Synthesis and structure of bis-(β-carotene) decanuclear Pd complexes

**Figure 2:**
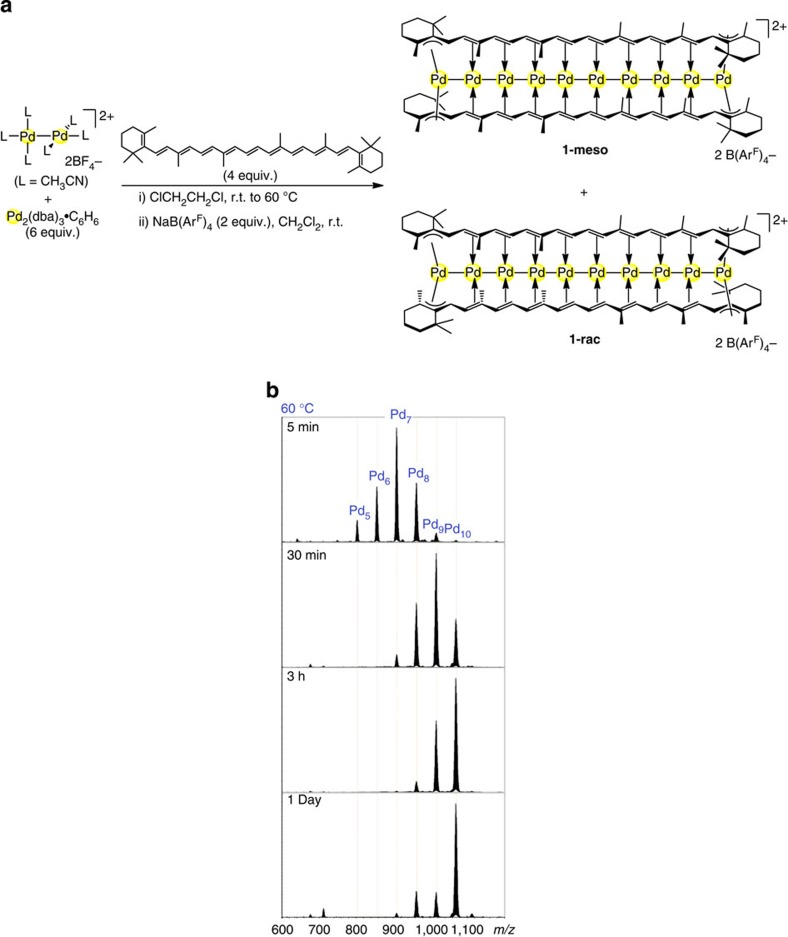
Synthesis of bis-(β-carotene) Pd_10_ chain complexes. (**a**) Synthesis of [Pd_10_(μ_10_-β-carotene)_2_][B(Ar^F^)_4_]_2_ (**1**), (**b**) ESI-MS monitoring of the formation of **1** at 60 °C showing the formation of metal-deficient intermediates [Pd_n_(β-carotene)_2_]^2+^ (*n*=5, 6, 7, 8 and 9).

**Figure 3:**
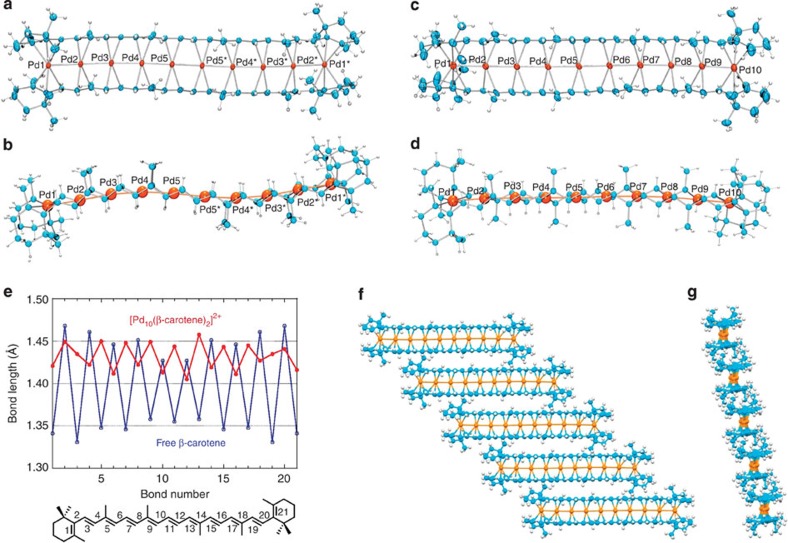
Structures of bis-(β-carotene) Pd_10_ chain complexes. (**a**) Thermal ellipsoid (50%) drawing of *meso*-[Pd_10_(μ_10_-β-carotene)_2_][B(Ar^F^)_4_]_2_ (**1-meso**). (**b**) Ball-stick drawing of **1-meso**. (**c**) Thermal ellipsoid (30%) drawing of *rac*-[Pd_10_(μ_10_-β-carotene)_2_][B(Ar^F^)_4_]_2_ (**1-rac**). (**d**) Ball-stick drawing of **1-rac**. (**e**) C–C bond lengths in **1-meso** and free β-carotene (CCDC-253816), determined by X-ray structural analyses. (**f**) A view of a part of an intermolecular backbone π–π stacking column of **1-meso** in the crystalline state. (**g**) A side view of a part of the π–π stacking column of **1-meso**. For **a**–**d**,**f**,**g**, B(Ar^F^)_4_ anions and non-coordinating solvent molecules were omitted for clarity.

We examined the homoleptic carotene–metal systems in which all auxiliary ligands contained in the starting metal complexes are replaced by carotene to afford a sandwich-type multimetal-binding motif. Pd was used because of its feasibility to undergo convergent metal assembly with the aids of relatively weak metal–ligand and metal–metal bonds^21^,^22^. At first, we investigated the full metalation of the bis-carotene π-framework. The redox–condensation reaction of [Pd_2_(CH_3_CN)_6_][BF_4_]_2_ (ref. 23) and excess Pd_2_(dba)_3_·C_6_H_6_ (ref. 24) in the presence of β-carotene at 60 °C gave the decanuclear palladium complex [Pd_10_(μ_10_-β-carotene)_2_][B(Ar^F^)_4_]_2_ (**1**, B(Ar^F^)_4_=B(3,5-(CF_3_)_2_C_6_H_3_)_4_) in 33% yield as a mixture of two isomers (Fig. 2a, 1**-meso**:**1-rac**=7:3). It is noted that use of Pd_2_(dba)_3_·CHCl_3_ instead of Pd_2_(dba)_3_·C_6_H_6_ gave a complicated mixture from which **1** was not obtained. The major isomer (**1-meso**) is poorly soluble in CH_3_CN, whereas another isomer (**1-rac**) is soluble, enabling us to separate the two isomers. The molecular structures of thus obtained yellow complexes **1-meso** and **1-rac** were determined by X-ray crystallographic analysis (Fig. 3). Remarkably, two β-carotene molecules flank an array of 10 Pd atoms through unprecedentedly large μ_10_-bridging π-coordination. The two β-carotene ligands are stacking in an eclipsed form in **1-meso** and in a staggered one in **1-rac**. The nine Pd–Pd bond lengths (2.5827(7)–2.7172(6) Å in **1-meso**; 2.6010(11)–2.7111(11) Å in **1-rac**) are shorter than that of bulk Pd (2.76 Å), indicating that 10 Pd atoms in **1-meso** or **1-rac** are connected through Pd–Pd bonds (Supplementary Table 1). The calculated indices of Mayer bond order for each Pd–Pd are ranged from 0.23 to 0.14 for **1-meso**; cf., for a typical Pd–Pd-bonded complex, Pd_2_Cl_2_(PH_3_)_4_, the index of Meyer bond order for Pd–Pd was calculated to be 0.60. The β-carotene ligands in **1-meso** showed reduced and inversed C=C/C–C bond length alternation, that is, the long/short alternation (1.46/1.41 Å) was found for the inner nonaene substructure in the β-carotene ligands in **1-meso**, being in between the C–C bond lengths in ethane (1.54 Å) and ethylene (1.34 Å; cf. 1.33/1.47 Å for the bond length alternation of free β-carotene; Fig. 3e). It is noted that the bis-β-carotene Pd_10_ chain dication of **1-meso** formed infinite intermolecular π–π stacking columns (the shortest intermolecular C···C distance is 3.51 Å) in the crystalline state (Fig. 3f,g). Such intermolecular backbone π–π stacking represents the typical property of the planar π-conjugated system. The dication of **1-rac** formed the π–π stacking dimer instead of the infinite column in the crystalline state. The ^1^H and ^13^C{^1^H} nuclear magnetic resonance (NMR) spectra of **1-meso** and **1-rac** in CD_2_Cl_2_ showed that all olefinic proton and carbon resonances of the β-carotene ligands appeared at the high-field region (olefinic moieties appeared at *δ*=3.5–2.6 ppm for ^1^H; *δ*=111–69 ppm for ^13^C), being consistent with the solid state structures determined by X-ray crystallography. The decanuclear sandwich complexes **1-meso** and **1-rac** are stable in solution even in the aerobic condition. Thus, it has been proven that bis-β-carotene π-framework can accommodate 10 Pd atoms array through remarkable multidentate bridging π-coordination. The decanuclear complexes **1-meso** and **1-rac** are the soluble and isolable organometallic clusters having a long metal chain. Existence of long inorganic palladium wires in solution was recently reported, where di- or tetranuclear palladium units are self-assembled through Pd–Pd interactions^25^,^26^. The extended (π-carbon framework)–(metal clusters) contact found in **1-meso** and **1-rac** may be related with the interface structure of the sp^2^-carbon material and metal clusters that is of current interests in materials science and catalysis^27^,^28^.

### Synthesis and structure of bis-(β-carotene) decanuclear PdPt complexes

To further explore the metal-binding ability of β-carotene, we next examined the binding of bimetallic chains by bis-β-carotene π-framework. It was difficult to obtain a single bimetallic chain product simply by using Pt_2_(dba)_3_ together with Pd_2_(dba)_3_ in the synthetic reaction. We then thought a stepwise synthesis, that is, if metal-deficient bis-β-carotene complexes [Pd_*n*_(β-carotene)_2_]^2+^ (*n*≤9) can be selectively constructed with Pd, subsequent incorporation of Pt may give bimetallic chains. The metal-deficient dications [Pd_*n*_(β-carotene)_2_]^2+^ (5≤*n*≤9) were indeed observed when the formation of **1** (Fig. 2a) was monitored by electrospray ionization mass spectroscopy (ESI-MS; Fig. 2b). Upon mixing the starting materials at ambient temperature, Pd_6_ and Pd_7_ complexes of β-carotene were detected as the major MS-detectable species after 3 h. Relatively small MS signals for Pd_5_, Pd_8_ and Pd_9_ complexes were also detected. Further incorporation of Pd into the bis-β-carotene framework proceeded gradually but was incomplete at ambient temperature after 2 days, resulting in that Pd_7_ and Pd_8_ complexes were the major MS-detectable species. Heating at 60 °C resulted in shift of the distribution of products to higher nuclearity species, eventually affording the Pd_10_ chain complexes as the major MS-detectable product. However, it has been difficult to isolate and characterize each of metal-deficient products from the reaction mixtures of the build-up reaction. We confirmed the existence of regioisomers for a short chain model, that is, [Pd_2_(1,10-diphenylpentaene)_2_][B(Ar^F^)_4_]_2_, which was obtained by the reaction of [Pd_2_(1,4-diphenyl-1,3-butadiene)_2_][BF_4_]_2_ (ref. 29) with 1,10-diphenyl-1,3,5,7,9-decapentaene at room temperature (r.t.), contained four regioisomers (57:27:10:6) as shown in Supplementary Fig. 1.

**Figure 4:**
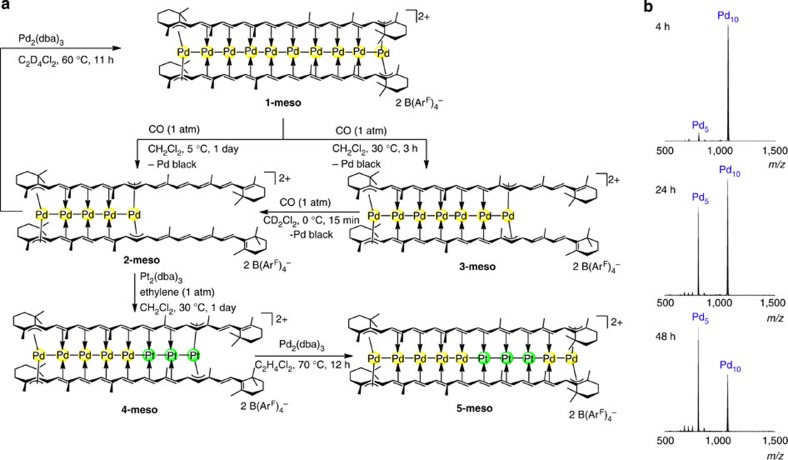
Synthesis of bimetallic PdPt chain sandwich complexes of β-carotene. (**a**) Demetalation and metalation of bis-β-carotene framework. Demetalation of **1-meso** with CO afforded the metal-deficient complex [Pd_5_(μ_5_-β-carotene)_2_][B(Ar^F^)_4_]_2_ (**2-meso**) or [Pd_7_(μ_7_-β-carotene)_2_][B(Ar^F^)_4_]_2_ (**3-meso**). Subsequent metalation of **2-meso** with Pt^0^ and then with Pd^0^ gave a mixed metal complex [Pd_5_Pt_3_(μ_8_-β-carotene)_2_][B(Ar^F^)_4_]_2_ (**4-meso**) and [Pd_5_Pt_3_Pd_2_(μ_10_-β-carotene)_2_][B(Ar^F^)_4_]_2_ (**5-meso**), respectively. (**b**) ESI-MS monitoring of the demetalation from **1-meso** at 0 °C under CO (1 atm) atmosphere.

**Figure 5:**
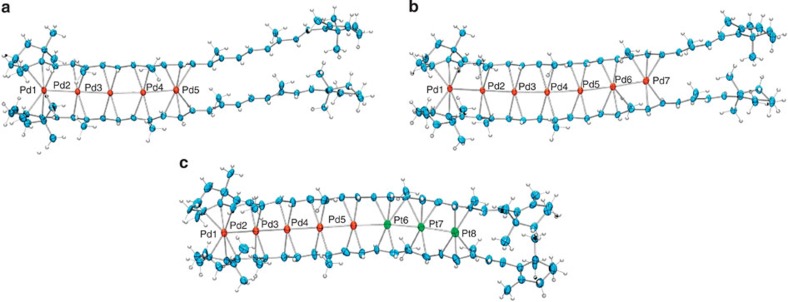
Structures of metal-deficient sandwich complexes of β-carotene. (**a**) Thermal ellipsoid (30%) drawing of [Pd_5_(μ_5_-β-carotene)_2_][B(Ar^F^)_4_]_2_ (**2-meso**). (**b**) Thermal ellipsoid (30%) drawing of [Pd_7_(μ_7_-β-carotene)_2_][B(Ar^F^)_4_]_2_ (**3-meso**). (**c**) Thermal ellipsoid (30%) drawing of [Pd_5_Pt_3_(μ_8_-β-carotene)_2_][B(Ar^F^)_4_]_2_ (**4-meso**). For **a**–**c**, B(Ar^F^)_4_ anions and non-coordinating solvent molecules were omitted for clarity. The coordination modes of two β-carotene ligands in the crystalline **3-meso** or **4-meso** are slightly different. Furthermore, in the crystal structures of the metal-deficient complexes, **2-meso**, **3-meso** and **4-meso**, the uncoordinated cyclohexenyl group are not superposed due to the rotation at C6–C7 bond. The NMR spectra of **2-meso**, **3-meso** or **4-meso** showed only a single set of β-carotene signals.

We then found that the metal-deficient complexes of β-carotene can be obtained as a single product by demetalation from the Pd_10_ complex **1-meso** with CO. Thus, the reaction of **1** with CO (1 atmosphere (atm)) at 5 °C for 1 day afforded [Pd_5_(β-carotene)_2_][B(Ar^F^)_4_]_2_ (**2-meso**) in 74% yield, together with a significant amount of Pd black (Fig. 4a). ^13^C{^1^H} NMR analysis as well as the single-crystal X-ray structure analysis of **2-meso** showed that the Pd_5_ chain occupied the half-part of the bis-β-carotene framework (Fig. 5a). The ESI-MS monitoring experiments on the demetalation reaction with CO (1 atm) at 0 °C showed that the starting Pd_10_ complex **1-meso** and the half-filled Pd_5_ complex **2-meso** were present as the major MS-detectable species during the reaction (Fig. 4b). The prolonged reactions for 1 week resulted in gradual increase of the MS signal for the Pd_4_ complex. The Pd_7_ complex [Pd_7_(β-carotene)_2_][B(Ar^F^)_4_]_2_ (**3-meso**) was also obtained by exposing **1-meso** to CO (1 atm) at 30 °C for 3 h in 20% yield (Fig. 4a). During this reaction, the major ESI-MS-detectable species were the starting Pd_10_ complex and the Pd_7_ complex (Supplementary Fig. 2), while the prolonged reactions resulted in further demetalation. The ^13^C{^1^H} NMR analysis as well as the single-crystal X-ray structure analysis (Fig. 5b) showed that the Pd_7_ chain was located in the bis-β-carotene framework. The demetalation of **3-meso** with CO (1 atm) at 0 °C occurred rapidly to afford **2-meso** with complete consumption of **3-meso** within 15 min. Thus, the pseudo-superposed β-carotene stacking structure was preserved during the demetalation under a CO atmosphere, giving a single regioisomer of metal-deficient sandwich. The results of the ESI-MS monitoring experiments suggested that the loss of a Pd^0^ atom from the Pd_10_ complex and from the Pd_5_ or Pd_7_ complex are relatively slow. The loss of Pd^0^ likely occurs from one end of the Pd chain, while it is not easy to explain the reason why the demetalation almost stopped at the Pd_5_ species or the Pd_7_ species in each reaction condition. There may be several factors that affect aggregation and dissociation of metals and organic ligands (for example, M–CO affinity^30^, M–carotene bond dissociation and M–M bond dissociation). In the case of associative ligand exchange, the relatively slow release of Pd^0^ from the filled Pd_10_ complex is probably due to the lower accessibility of the terminal Pd atoms, which are sterically hindered by the bulky terminal β-groups of the β-carotene ligands, to CO. Consistently, demetalation from the Pd_7_ complex, where one end of the Pd_7_ chain is less hindered by ligands, occurred much faster at 0 °C than that from the Pd_10_ complex.

We next confirmed that metal-refilling reaction from isolated metal-deficient complexes proceeds smoothly by using the Pd_5_ complex **2-meso**, that is, addition of Pd_2_(dba)_3_·C_6_H_6_ to **2-meso** in C_2_D_4_Cl_2_ at 60 °C afforded the Pd_10_ complex **1-meso** (41% yield). We then tested whether metal refilling of **2-meso** with Pt^0^ is possible. Thus, the bimetallic Pd_5_Pt_3_ chain complex [Pd_5_Pt_3_(β-carotene)_2_][B(Ar^F^)_4_]_2_ (**4-meso**) was formed by treatment of **2-meso** with Pt_2_(dba)_3_·CHCl_3_ in the presence of ethylene (1 atm) at 30 °C for 1 day (59% yield; Fig. 4a). In the absence of ethylene, the metalation with Pt_2_(dba)_3_ did not proceed at the present condition. Addition of ethylene generated Pt–ethylene complexes *in situ*, which might be more reactive and soluble than Pt_2_(dba)_3_ (ref. 31). The Pd_5_−Pt_3_ mixed metal arrangement in **4-meso** was confirmed by X-ray crystallographic analysis (Fig. 5c). The Pd_5_ chain (Pd–Pd=2.731(2)–2.629(2) Å) and the Pt_3_ chain (Pt–Pt=2.6612(9) and 2.6864(10) Å) are connected through Pd–Pt bond (2.657(2) Å). The Pd_5_Pt_3_ arrangement was also confirmed by ^13^C NMR analyses in CD_2_Cl_2_ where only one set of β-carotene signals was observed at 25 °C, and ^13^C signals for Pt-bound carbons appeared at relatively higher field compared with those for Pd-bound carbons. Further metalation of **4-meso** with Pd_2_(dba)_3_·C_6_H_6_ at 70 °C gave decanuclear bimetallic chain complex [Pd_5_Pt_3_Pd_2_(β-carotene)_2_][B(Ar^F^)_4_]_2_ (**5-meso**) (Fig. 4a). The alternative metal arrangement, Pd_5_–Pt_3_–Pd_2_ was confirmed by assignment of the upfield shifted Pt-bound carbons of the β-carotene ligands in ^13^C{^1^H} NMR analyses in CD_2_Cl_2_, that is, the substantial upfield shifts of the Pt-bound carbons of the β-carotene ligands in **5-meso** relative to those in **1-meso** (*Δδ*=8–13 ppm) were observed, while the chemical shifts of the Pd-bound carbons in **5-meso** are similar to those in **1-meso** (*Δδ*=0–2 ppm). Thus, β-carotene has the ability to bind bimetallic decanuclear chain, where stepwise demetalation–metalation sequence is useful in controlling the bimetal arrangement. Such reversible accommodation/liberation of multinuclear metal atoms has rarely been attained in metal cluster chemistry^32^, representing a facile dynamic metal-binding feature derived from weakly coordinating olefin π-coordination^33^,^34^ as well as M–M bonds in organometallic sandwich frameworks^35^.

### Absorption spectra of bis-(β-carotene) Pd complexes

It is noted that [Pd_*n*_(β-carotene)_2_][B(Ar^F^)_4_]_2_ showed a nuclearity-dependent absorption profile, that is, the red-shift of maximum absorption bands was observed according to decrease of the number of Pd atoms (362 nm for **1-meso**, 373 nm for **3-meso** and 468 nm for **2-meso**) (Supplementary Fig. 3). The absorption spectra were well described by the time-dependent density functional theory calculations at the Coulomb-attenuated B3LYP level^36^, suggesting that (i) the absorption bands originate mainly from the ligand-to-metal charge transfer (Supplementary Fig. 4 and Supplementary Table 2) and (ii) the observed red-shift with decreasing number of Pd atoms reflects the increasing stabilization of lower-lying unoccupied molecular orbitals that have antibonding character for *d*σ(M)-*d*σ(M) and *d*(M)-*p*(L) orbital interactions (Supplementary Figs 5–7 and Supplementary Tables 3–5). Visible-light irradiation resulted in the formation of **1** without heating, that is, the reaction similar to Fig. 2a with visible light irradiation (Xenon lamp, >385 nm) yielded **1** at 20 °C in 24% yield (**1-meso**:**1-rac**=7:3) (Supplementary Fig. 8 and Supplementary Methods).

## Discussion

In this report, it has been proven that β-carotene, a naturally abundant and readily available unsaturated hydrocarbon pigment, has the ability to bind decanuclear homo- and heterometal chains through unprecedentedly large μ_10_-bridging π-coordination. The present results showed that natural extended π-conjugated unsaturated hydrocarbons can be utilized as the multidentate π-scaffolds for the construction of giant metal clusters. Future studies will focus on the physical and chemical properties of the rod-like bis-carotene decametal chain sandwich complexes, such as self-assembling behaviour, multielectron redox behaviour and charge mobility.

## Methods

### Synthesis and characterization of compounds

All manipulations were conducted under a nitrogen atmosphere using standard Schlenk or drybox techniques. The β-carotene–metal complexes were characterized by elemental analyses, ESI-MS analyses and NMR. The assignment of each resonance in NMR analysis was made with aid of heteronuclear single-quantum correlation (HSQC) or heteronuclear multiple-quantum correlation (HMQC) and heteronuclear multiple-bond correlation (HMBC) techniques. Furthermore, the five complexes (**1-meso**, **1-rac**, **2-meso**, **3-meso** and **4-meso**) were structurally determined by X-ray crystallographic analyses (Supplementary Data 1, 2, 3, 4, 5).

### Synthesis of [Pd_10_(β-carotene)_2_][B(Ar^F^)_4_]_2_ (1-meso) and (1-rac)

To a suspension of β-carotene (679 mg, 1.27 mmol) in ClCH_2_CH_2_Cl (200 ml) were added Pd_2_(dba)_3_·(C_6_H_6_) (1.89 g, 1.90 mmol) and [Pd_2_(CH_3_CN)_6_][BF_4_]_2_ (200 mg, 0.316 mmol) at r.t. The reaction mixture was stirred under nitrogen atmosphere at 60 °C for 1 day. The reaction mixture was filtered and the filtrate was dried *in vacuo*. The obtained brown powder and NaB(Ar^F^)_4_ (560 mg, 0.632 mmol) was added to CH_2_Cl_2_, and the mixture was stirred for 5 min at r.t. Et_2_O was added to the solution and the mixture was filtered. The filtrate was dried *in vacuo* to yield a red powder. After washing with CH_3_CN, [Pd_10_(β-carotene)_2_][B(Ar^F^)_4_]_2_ (**1-meso**) was isolated as an yellow powder (290 mg, 24%). [Pd_10_(β-carotene)_2_][B(Ar^F^)_4_]_2_ (**1-rac**) was obtained by recrystallization from the CH_3_CN solution (107 mg, 9%). For **1-meso**: ^1^H NMR (400 MHz, CD_2_Cl_2_, 25 °C): *δ*–0.27 (s, 12H), –0.15 (s, 12H), 0.33 (s, 12H), 1.54 (s, 12H), 1.64 (m, 4H), 1.78 (m, 4H), 1.99 (m, 4H), 2.08 (m, 4H), 2.10 (s, 12H), 2.65 (d, *J*=12 Hz, 4H), 2.66 (d, *J*=12 Hz, 4H), 2.86 (dd, *J*=3 Hz, *J*=9 Hz, 4H), 2.95 (d, *J*=12 Hz, 4H), 3.04–3.11 (m, 8H), 3.28–3.45 (m, 8H), 3.47 (t, *J*=12 Hz, 4H), 7.50 (s, 8H, *p*-*B(Ar*^*F*^)_*4*_), 7.65 (s, 16H, *o-B(Ar*^*F*^)_*4*_). ^13^C NMR (100 MHz, CD_2_Cl_2_, 25 °C): *δ* 14.3, 15.0, 20.5, 23.7, 28.1, 31.8, 34.9, 36.8, 43.5, 74.1, 74.7, 80.7, 82.1, 83.0, 83.3, 84.5, 98.0, 103.3, 109.7, 110.1, 117.8 (*p-B(Ar*^*F*^)_*4*_), 125.4 (*CF*_*3*_*-B(Ar*^*F*^)_*4*_), 129.2 (*m-B(Ar*^*F*^)_*4*_), 135.1 (*o-B(Ar*^*F*^)_*4*_), 162.1 (*ipso-B(Ar*^*F*^)_*4*_). MS (ESI) *m/z* calcd. for [C_80_H_112_Pd_10_]^2+^: 1,068.9601, found: 1,068.9647. Anal. calcd. For C_144_H_136_B_2_F_48_Pd_10_: C, 44.76; H, 3.55, found: C, 44.77; H, 3.73. A single crystal suitable for X-ray crystallographic analysis was grown from a CH_2_Cl_2_-Toluene solution (Supplementary Methods). For **1-rac**: ^1^H NMR (400 MHz, CD_2_Cl_2_, 25 °C): *δ*–0.72 (s, 12H), 0.20 (s, 12H), 0.38 (s, 12H), 1.40 (m, 4H), 1.70 (m, 4H), 1.73 (s, 12H), 1.80 (m, 4H), 1.83 (s, 12H), 2.08 (m, 4H), 2.77 (d, *J*=12 Hz, 4H), 2.80 (d, *J*=12 Hz, 4H), 2.85 (d, *J*=12 Hz, 4H), 3.03 (d, *J*=12 Hz, 4H), 3.07 (dd, *J*=3 Hz, *J*=9 Hz, 4H), 3.33 (m, 4H), 3.34 (t, *J*=12 Hz, 4H), 3.46 (dd, *J*=3 Hz, *J*=9 Hz, 4H), 3.52 (m, 4H), 7.30 (s, 8H, *p*-*B(Ar*^*F*^)_*4*_), 7.65 (s, 16H, *o-B(Ar*^*F*^)_*4*_). ^13^C NMR (100 MHz, CD_2_Cl_2_, 25 °C): *δ* 13.7, 13.8, 20.0, 24.4, 29.4, 29.7, 34.4, 36.5, 43.1, 69.3, 76.5, 79.5, 84.0, 87.5, 88.1, 89.3, 95.0, 96.1, 99.4, 108.1, 117.8 (*p-B(Ar*^*F*^)_*4*_), 125.4 (*CF*_*3*_*-B(Ar*^*F*^)_*4*_), 129.2 (*m-B(Ar*^*F*^)_*4*_), 135.1 (*o-B(Ar*^*F*^)_*4*_), 162.1 (*ipso-B(Ar*^*F*^)_*4*_). MS (ESI) *m/z* calcd. for [C_80_H_112_Pd_10_]^2+^: 1,068.9601, found: 1,068.9432. Anal. calcd. For C_144_H_136_B_2_F_48_Pd_10_·C_6_H_14_: C, 45.60; H, 3.83, found: C, 45.48; H, 3.99. A single crystal suitable for X-ray crystallographic analysis was grown from a diethylether−hexane solution (Supplementary Methods).

### Synthesis of [Pd_5_(β-carotene)_2_][B(Ar^F^)_4_]_2_ (2-meso)

CO gas (1 atm) was bubbled in a CH_2_Cl_2_ solution (220 ml) of [Pd_10_(β-carotene)_2_][B(Ar^F^)_4_]_2_ (**1-meso**) (340 mg, 88.0 μmol) at 5 °C for 24 h. The reaction mixture was filtered and the filtrate was dried *in vacuo* to give a dark brown powder. After extraction with CH_3_CN, the volatiles were removed in vacuo to give [Pd_5_(β-carotene)_2_][B(Ar^F^)_4_]_2_ (**2-meso**) as a dark brown powder (218 mg, 74%). ^1^H NMR (400 MHz, CD_2_Cl_2_, 25 °C): δ–0.29 (s, 6H), 0.05 (s, 6H), 0.90 (s, 6H), 1.05 (s, 6H), 1.06 (s, 6H), 1.47 (m, 4H), 1.60 (s, 6H), 1.60–1.68 (m, 8H), 1.74 (s, 6H), 1.92–2.08 (m, 6H), 1.94 (s, 6H), 1.98 (s, 6H), 2.02 (d, *J*=12 Hz, 2H), 2.06 (s, 6H), 2.12 (m, 2H), 2.39 (d, *J*=12 Hz, 2H), 2.52 (d, *J*=12 Hz, 2H), 2.92 (t, *J*=12 Hz, 2H), 2.96 (t, *J*=12 Hz, 2H), 3.3 (m, 4H), 3.67 (t, *J*=12 Hz, 2H), 5.13 (t, *J*=12 Hz, 2H), 5.85 (t, *J*=12 Hz, 2H), 6.10 (d, *J*=12 Hz, 2H), 6.15 (d, *J*=16 Hz, 2H), 6.22 (d, *J*=12 Hz, 2H), 6.26 (d, *J*=16 Hz, 2H), 6.29 (d, *J*=16 Hz, 2H), 6.91 (dd, *J*=12 Hz, *J*=16 Hz, 2H), 7.49 (s, 8H, *p*-*B(Ar*^*F*^)_*4*_), 7.64 (s, 16H, *o-B(Ar*^*F*^)_*4*_). ^13^C NMR (100 MHz, CD_2_Cl_2_, 25 °C): *δ* 13.0, 13.3, 14.0, 14.2, 19.6, 19.9, 22.1, 24.0, 28.0, 29.2, 29.4, 31.6, 33.7, 34.6, 35.0, 36.8, 40.1, 42.7, 76.8, 78.9, 79.7, 89.2, 89.4, 89.6, 91.8, 92.5, 103.5, 111.8, 112.3, 117.8 (*p-B(Ar*^*F*^)_*4*_), 123.4, 125.5 (*CF*_*3*_*-B(Ar*^*F*^)_*4*_), 126.7, 129.2 (*m-B(Ar*^*F*^)_*4*_), 129.3, 129.9, 130.4, 130.6, 135.1 (*o-B(Ar*^*F*^)_*4*_), 135.7, 137.6, 138.0, 140.3, 143.9, 162.1 (*ipso-B(Ar*^*F*^)_*4*_). MS (ESI) *m/z* calcd. for [C_80_H_112_Pd_5_]^2+^: 802.6996, found: 802.7014. Anal. calcd. For C_144_H_136_B_2_F_48_Pd_5_·C_6_H_6_: C, 52.83; H, 4.20, found: C, 52.65; H, 4.35. A single crystal suitable for X-ray crystallographic analysis was grown from a diethylether–benzene solution (Supplementary Methods).

### Synthesis of [Pd_7_(β-carotene)_2_][B(Ar^F^)_4_]_2_ (3-meso)

CO gas (1 atm) was bubbled in a CH_2_Cl_2_ solution (100 ml) of [Pd_10_(β-carotene)_2_][B(Ar^F^)_4_]_2_ (**1-meso**) (107 mg, 27.8 μmol) at 30 °C for 3 h in the dark. The reaction mixture was filtered and the filtrate was dried *in vacuo* to give a dark red powder. The resultant powder was washed with CH_3_CN and dried in vacuo. Et_2_O was added and the mixture was filtered. The filtrate was dried *in vacuo* to give [Pd_7_(β-carotene)_2_][B(Ar^F^)_4_]_2_ (**3-meso**) as a red powder (20.0 mg, 20%). ^1^H NMR (400 MHz, CD_2_Cl_2_, 25 °C): δ–0.27 (s, 6H), –0.04 (s, 6H), 0.07 (s, 6H), 0.67 (s, 6H), 1.12 (s, 6H), 1.13 (s, 6H), 1.51 (m, 4H), 1.59 (s, 6H), 1.61–1.70 (m, 6H), 1.79 (s, 6H), 1.97 (m, 2H), 2.06 (s, 6H), 2.06–2.15 (m, 8H), 2.23 (d, *J*=12 Hz, 2H), 2.33 (d, *J*=12 Hz, 2H), 2.34 (s, 6H), 2.40 (t, *J*=12 Hz, 2H), 2.79 (d, *J*=12 Hz, 2H), 2.85 (d, *J*=12 Hz, 2H), 3.03 (d, *J*=12 Hz, 2H), 3.27 (d, *J*=12 Hz, 2H), 3.34 (m, 4H), 3.56 (t, *J*=12 Hz, 2H), 3.65 (d, *J*=12 Hz, 2H), 4.50 (d, *J*=12 Hz, 2H), 6.08 (d, *J*=12 Hz, 2H), 6.28 (d, *J*=16 Hz, 2H), 6.44 (d, *J*=16 Hz, 2H), 6.46 (t, *J*=12 Hz, 2H), 7.48 (s, 8H, *p*-*B(Ar*^*F*^)_*4*_), 7.63 (s, 16H, *o-B(Ar*^*F*^)_*4*_). ^13^C NMR (100 MHz, CD_2_Cl_2_, 25 °C): *δ* 13.6, 14.4, 14.6, 14.7, 19.6, 20.2, 22.2, 23.9, 28.0, 29.3, 29.5, 31.7, 34.0, 34.5, 34.9, 36.7, 40.2, 43.0, 75.5, 76.8, 79.0, 80.3, 80.6, 86.7, 87.9, 88.8, 89.2, 95.5, 96.1, 96.4, 100.9, 103.5, 111.2, 117.1, 117.8 (*p-B(Ar*^*F*^)_*4*_), 125.5 (*CF*_*3*_*-B(Ar*^*F*^)_*4*_), 127.2, 129.2 (*m-B(Ar*^*F*^)_*4*_), 129.4, 131.5, 135.1 (*o-B(Ar*^*F*^)_*4*_), 136.6, 137.6, 140.4, 162.1 (*ipso-B(Ar*^*F*^)_*4*_). MS (ESI) *m/z* calcd. for [C_80_H_112_Pd_7_]^2+^: 909.1038, found: 909.1082. Anal. calcd. For C_144_H_136_B_2_F_48_Pd_7_·(C_6_H_6_)_2_: C, 50.62; H, 4.03, found: C, 50.73; H, 4.06. A single crystal suitable for X-ray crystallographic analysis was grown from a diethylether–benzene solution (Supplementary Methods).

### Synthesis of [Pd_5_Pt_3_(β-carotene)_2_][B(Ar^F^)_4_]_2_ (4-meso)

Ethylene gas (1 atm) was bubbled in a CH_2_Cl_2_ solution (30 ml) of [Pd_5_(β-carotene)_2_][B(Ar^F^)_4_]_2_ (**2-meso**) (54.0 mg, 16.2 μmol) and Pt_2_(dba)_3_**·**(CHCl_3_) (100 mg, 82.5 μmol) at r.t. for 5 min. After the solution was stirred at 30 °C for 1 day, the colour turned dark brown. The reaction mixture was dried *in vacuo* to give a dark brown powder. After extraction with Et_2_O and washing with C_6_H_6_, [Pd_5_Pt_3_(β-carotene)_2_][B(Ar^F^)_4_]_2_ (**4-meso**) was isolated as a yellow solid (37.6 mg, 59%). ^1^H NMR (400 MHz, CD_2_Cl_2_, 25 °C): *δ*–0.28 (s, 6H), –0.11 (s, 6H), 0.50 (s, 12H), 1.17 (s, 6H), 1.21 (s, 6H), 1.56 (s, 6H), 1.56–1.59 (m, 4H), 1.60–1.72 (m, 8H), 1.84 (s, 6H), 1.98 (m, 2H), 2.01 (s, 6H), 2.07 (s, 6H), 2.09 (m, 2H), 2.13–2.18 (m, 4H), 2.34 (d, *J*=12 Hz, 2H), 2.36 (d, *J*=12 Hz, 2H), 2.43 (t, *J*=12 Hz, 2H), 2.71 (d, *J*=12 Hz, 2H), 2.76 (d, *J*=14 Hz, 2H), 2.81 (d, *J*=12 Hz, 2H), 3.00 (t, *J*=11 Hz, 2H), 3.04 (d, *J*=12 Hz, 2H), 3.11 (d, *J*=12 Hz, 2H), 3.3 (m, 4H), 3.40 (m, 2H), 3.48 (t, *J*=12 Hz, 2H), 3.79 (d, *J*=11 Hz, 2H), 6.47 (d, *J*=16 Hz, 2H), 6.68 (d, *J*=16 Hz, 2H), 7.49 (s, 8H, *p*-*B(Ar*^*F*^)_*4*_), 7.65 (s, 16H, *o-B(Ar*^*F*^)_*4*_). ^13^C NMR (100 MHz, CD_2_Cl_2_, 25 °C): *δ* 14.3, 14.6, 15.5, 17.5, 19.7, 20.3, 22.2, 23.8, 28.0, 29.2, 29.7, 31.7, 33.9, 34.7, 34.9, 36.8, 40.1, 43.2, 64.4, 67.3, 74.9, 75.1, 75.9, 78.1, 78.7, 79.9, 80.6, 85.2, 85.7, 87.6, 96.9, 98.4, 98.6, 103.5, 110.6, 114.4, 117.8 (*p-B(Ar*^*F*^)_*4*_), 125.5 (*CF*_*3*_*-B(Ar*^*F*^)_*4*_), 129.1, 129.2 (*m-B(Ar*^*F*^)_*4*_), 132.3, 135.1 (*o-B(Ar*^*F*^)_*4*_), 136.0, 136.5, 162.1 (*ipso-B(Ar*^*F*^)_*4*_). MS (ESI) *m/z* calcd. for [C_80_H_112_Pd_5_Pt_3_]^2+^: 1,095.1460, found: 1095.1291. Anal. calcd. For C_144_H_136_B_2_F_48_Pd_5_Pt_3_·(C_6_H_6_)_2_: C, 45.99; H, 3.66, found: C, 46.10; H, 3.86. A single crystal suitable for X-ray crystallographic analysis was grown from a dichloromethane–benzene solution (Supplementary Methods).

### Synthesis of [Pd_5_Pt_3_Pd_2_(β-carotene)_2_][B(Ar^F^)_4_]_2_ (5-meso)

To a solution of [Pd_5_Pt_3_(β-carotene)_2_][B(Ar^F^)_4_]_2_ (**4-meso**) (58.0 mg, 14.8 μmol) in ClCH_2_CH_2_Cl (50 ml) was added Pd_2_(dba)_3_·(C_6_H_6_) (200 mg, 201 μmol), and the reaction mixture was stirred under nitrogen atmosphere at 70 °C for 12 h. The mixture was filtered and the filtrate was dried *in vacuo*. After reprecipitation with CH_2_Cl_2_/hexane, the yellow powder was obtained. After drying *in vacuo*, the product was analysed by NMR, showing that [Pd_5_Pt_3_Pd_2_(β-carotene)_2_][B(Ar^F^)_4_]_2_ (**5-meso**) was formed as a major product with an unidentified minor product (major:minor=8:2; a mixture of two products: 43 mg). For **5-meso**: ^1^H NMR (600 MHz, CD_2_Cl_2_, 25 °C): *δ*–0.27 (s, 6H), –0.22 (s, 6H), –0.17 (s, 6H), 0.11 (s, 6H), 0.31 (s, 6H), 1.01 (s, 6H), 1.48 (s, 6H), 1.54 (s, 6H), 1.63 (m, 4H), 1.78 (m, 4H), 1.98 (m, 4H), 2.05 (s, 6H), 2.08 (m, 4H), 2.09 (s, 6H), 2.52 (d, *J*=12 Hz, 2H), 2.56 (d, *J*=12 Hz, 2H), 2.60 (t, *J*=12 Hz, 2H), 2.62 (d, *J*=12 Hz, 2H), 2.66 (d, *J*=12 Hz, 2H), 2.74 (d, *J*=12 Hz, 2H), 2.90 (d, *J*=12 Hz, 4H), 2.91 (d, *J*=12 Hz, 2H), 2.95 (d, *J*=12 Hz, 2H), 3.07 (d, *J*=12 Hz, 2H), 3.19 (t, *J*=12 Hz, 2H), 3.30 (m, 8H), 3.40 (t, *J*=12 Hz, 2H), 3.42 (t, *J*=12 Hz, 2H), 7.51 (s, 8H, *p*-*BAr*^*F*^_*4*_), 7.66 (s, 16H, *o-BAr*^*F*^_*4*_). ^13^C NMR (150 MHz, CD_2_Cl_2_, 25 °C): *δ* 14.1, 14.3, 14.8, 15.8, 20.4, 20.5, 23.7, 28.1, 28.2, 31.8, 31.9, 34.9, 36.8, 36.9, 43.4, 43.5, 70.0, 71.1, 71.2, 71.3, 71.8, 73.6, 74.1, 74.6, 74.8, 80.5, 81.8, 83.1, 83.4, 84.6, 89.9, 98.2, 103.5, 105.3, 109.8, 110.4, 111.4, 117.9 (*p-BAr*^*F*^_*4*_), 125.0 (*CF*_*3*_*-BAr*^*F*^_*4*_), 129.2 (*m-BAr*^*F*^_*4*_), 135.2 (*o-BAr*^*F*^_*4*_), 162.1 (*ipso-BAr*^*F*^_*4*_). MS (ESI) *m/z* calcd. for [C_80_H_112_Pd_7_Pt_3_]^2+^: 1,201.5503, found: 1,201.5553. Anal. calcd. For C_144_H_136_B_2_F_48_Pd_7_Pt_3_·(C_6_H_6_): C, 42.81; H, 3.40, found: C, 42.87; H, 3.46. The sample suitable for elemental analysis was obtained from a dichloromethane–benzene solution. The minor product might be an isomer of **5-meso** having a different metal arrangement (Supplementary Methods).

### Synthesis of [Pd_2_(1,10-diphenylpentaene)_2_][B(Ar^F^)_4_]_2_

To a suspension of 1,10-diphenyl-1,3,5,7,9-decapentaene (80.0 mg, 0.28 mmol) in CH_2_Cl_2_ (30 ml) was added [Pd_2_(1,4-diphenyl-1,3-butadiene)][B(Ar^F^)_4_]_2_ (299.6 mg, 0.13 mmol). The mixture was stirred for 1 h at r.t. The reaction mixture was filtered and poured into hexane to give deep-green precipitation. [Pd_2_(1,10-diphenylpentaene)_2_][B(Ar^F^)_4_]_2_was isolated as a mixture of four isomers by recrystallization (262.1 mg, 82%, isomer ratio 57:27:10:6). ^1^H NMR (600 MHz, CD_2_Cl_2_, 25 °C) of vinyl protons in **A** (57% of products): *δ* 3.30 (dd, *J*=12 Hz, *J*=12 Hz, 1H), 3.38 (dd, *J*=12 Hz, *J*=12 Hz, 1H), 3.54 (dd, *J*=12 Hz, *J*=12 Hz, 1H), 3.75 (dd, *J*=11 Hz, *J*=12 Hz, 1H), 4.29 (dd, *J*=11 Hz, *J*=13 Hz, 1H), 4.33 (dd, *J*=11 Hz, *J*=12 Hz, 1H), 4.48 (dd, *J*=12 Hz, *J*=13 Hz, 1H), 5.07 (dd, *J*=12 Hz, *J*=14 Hz, 1H), 5.52 (dd, *J*=12 Hz, *J*=13 Hz, 1H), 5.77 (dd, *J*=11 Hz, *J*=13 Hz, 1H), 5.90 (dd, *J*=11 Hz, *J*=15 Hz, 1H), 6.05 (dd, *J*=12 Hz, *J*=14 Hz, 1H), 6.08 (dd, *J*=11 Hz, *J*=15 Hz, 1H), 6.14 (d, *J*=14 Hz, 1H), 6.21 (dd, *J*=11 Hz, *J*=15 Hz, 1H), 6.40 (dd, *J*=11 Hz, *J*=11 Hz, 1H), 6.47 (dd, *J*=11 Hz, *J*=14 Hz, 1H), 6.50 (d, *J*=15 Hz, 1H), 6.69 (d, *J*=15 Hz, 1H), 6.71 (dd, *J*=15 Hz, 1H). **B** or **C** (27% of products): *δ* 3.34 (dd, *J*=11 Hz, *J*=11 Hz, 2H), 3.69 (dd, *J*=11 Hz, *J*=11 Hz, 2H), 4.32 (dd, *J*=11 Hz, *J*=13 Hz, 2H), 4.98 (dd, *J*=11 Hz, *J*=14 Hz, 2H), 5.46 (dd, *J*=12 Hz, *J*=14 Hz, 2H), 5.76 (dd, *J*=12 Hz, 13 Hz, 2H), 6.21 (dd, *J*=11 Hz, *J*=14 Hz, 2H), 6.42 (d, *J*=14 Hz, 2H), 6.61 (dd, *J*=11 Hz, *J*=15 Hz, 2H), 6.80 (dd, *J*=15 Hz, 2H). **B** or **C** (10% of products): *δ* 3.30 (dd, *J*=11 Hz, *J*=11 Hz, 2H), 3.78 (dd, *J*=11 Hz, *J*=11 Hz, 2H), 4.23 (dd, *J*=11 Hz, *J*=13 Hz, 2H), 5.03 (dd, *J*=11 Hz, *J*=14 Hz, 2H), ca. 5.46 (2H), 6.13 (d, *J*=14 Hz, 2H), 6.31 (dd, *J*=11 Hz, *J*=15 Hz, 2H), ca. 6.51 (2H), 6.56 (dd, *J*=11 Hz, *J*=14 Hz, 2H), 7.01 (dd, *J*=15 Hz, 2H). **D** (6% of products): *δ* 3.48 (m, 4H), 4.49 (m, 4H), 6.01 (dd, *J*=11 Hz, *J*=13 Hz, 4H), 6.18 (dd, *J*=11 Hz, *J*=15 Hz, 4H), 6.69 (d, *J*=15.6 Hz, 4H). Anal. calcd. For C_108_H_64_B_2_F_48_Pd_2_: C, 51.72; H, 2.57, found: C, 51.59; H, 2.58 (Supplementary Methods).

## Author contributions

The idea and plans of this research were made by T.Mu. Experiments and data analysis were performed by S.H., Y.T., M.Y., K.Y., K.M., K.T., T.Ma., S.K. and T.Mu. The theoretical calculations were performed by Y.K. and T.Y. The manuscript was co-written by T.Mu., Y.K. and T.Y. All authors discussed the results.

## Additional information

**Accession codes:** The X-ray crystallographic coordinates for structures reported in this Article have been deposited at the Cambridge Crystallographic Data Centre (CCDC), under deposition numbers CCDC 1001723-1001727. These data can be obtained free of charge from The CCDC via www.ccdc.cam.ac.uk/data_request/cif.

**How to cite this article:** Horiuchi, S. *et al*. Multinuclear metal-binding ability of a carotene. *Nat. Commun*. 6:6742 doi: 10.1038/ncomms7742 (2015).

## Supplementary Material

Supplementary Information Supplementary Figures 1-7, Supplementary Tables 1-2, Supplementary Methods and Supplementary References

Supplementary Data 1 Crystallographic Information File for 1-meso.

Supplementary Data 2 Crystallographic Information File for 1-rac.

Supplementary Data 3 Crystallographic Information File for 2-meso.

Supplementary Data 4 Crystallographic Information File for 3-meso.

Supplementary Data 5 Crystallographic Information File for 4-meso.

## Figures and Tables

**Figure 1:**
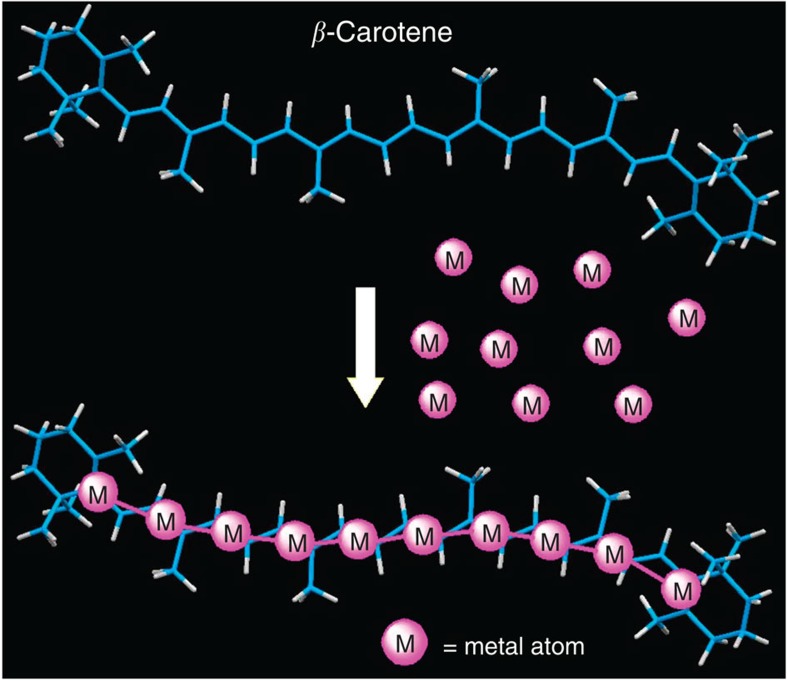
Schematic representation of multinuclear metal binding by β-carotene. Many metal atoms are assembled on the π-conjugated plane of β-carotene.
