# Supplementary material for: Multinuclear metal-binding ability of a carotene
Source: Nat Commun. 2015 Apr 10;6:6742. doi: 10.1038/ncomms7742 (PMC4403383; doi:10.1038/ncomms7742)
Supplement: Supplementary Information — Supplementary Figures 1-7, Supplementary Tables 1-2, Supplementary Methods and Supplementary References [file ncomms7742-s1.pdf]

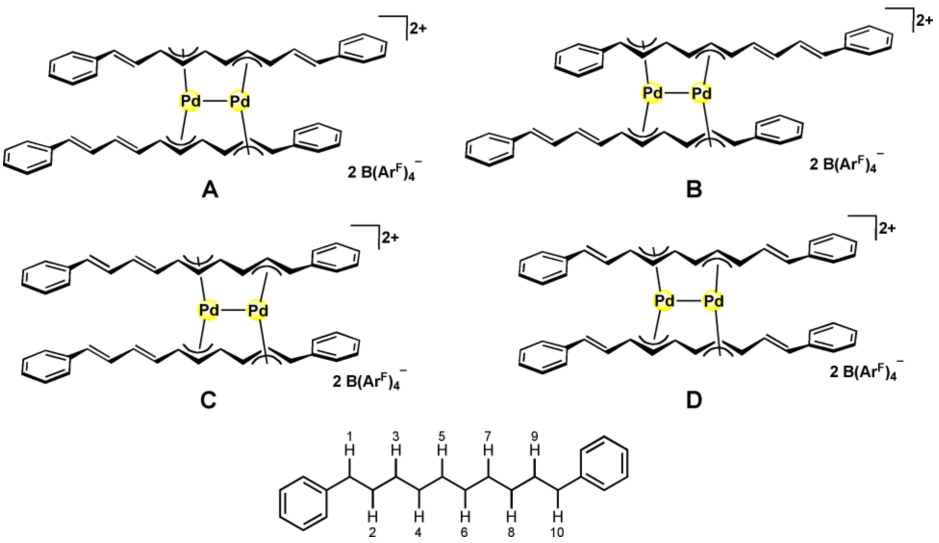

Supplementary Figure 1. Four isomers of $[Pd_2(1,10\text{-diphenylpentaene})_2][B(Ar^F)_4]_2$.

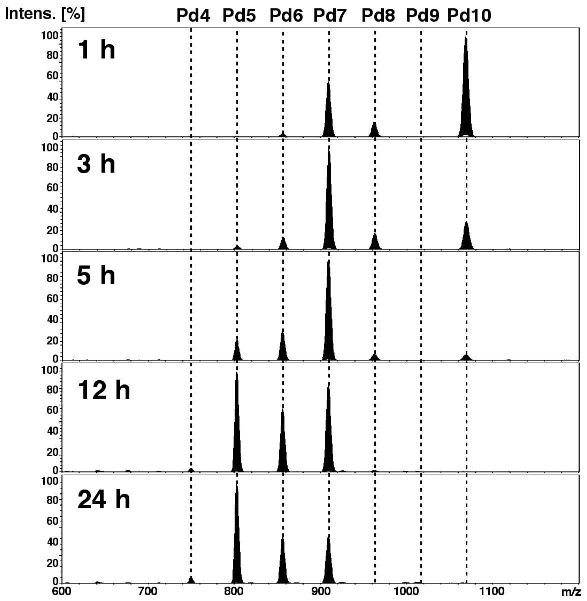

Supplementary Figure 2. ESI-MS monitoring of the demetalation from 1-meso at 30 °C under CO (1 atm) atmosphere.

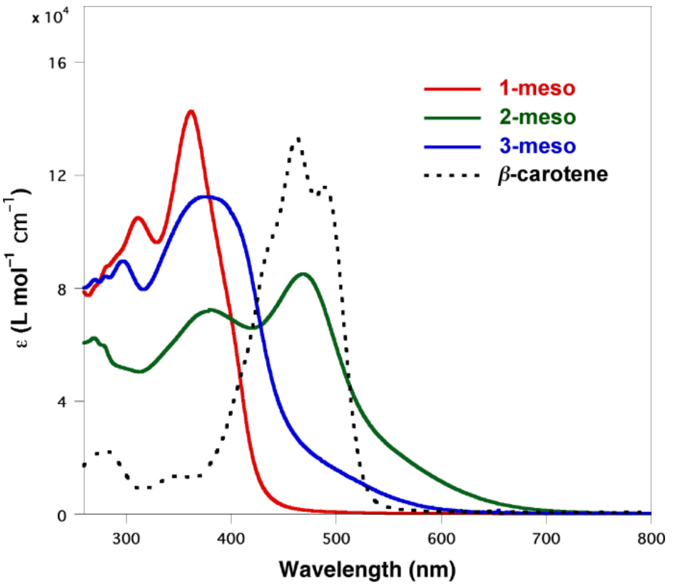

Supplementary Figure 3. UV-Vis spectra of $[Pd_n(\beta\text{-carotene})_2][B(Ar^F)_4]_2$ (n = 10, 7, 5) and free *β*-carotene in CH_2Cl_2 at 25 ºC.

$[Pd_5(\beta\text{-carotene})_2]^{2+}$

A

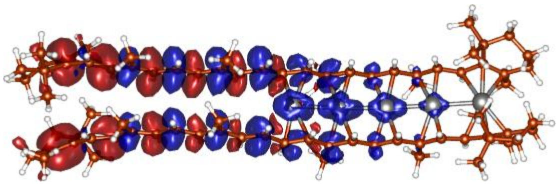

B

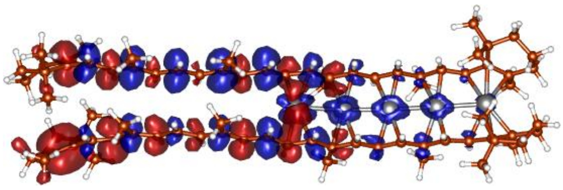

$[Pd_7(\beta\text{-carotene})_2]^{2+}$

C

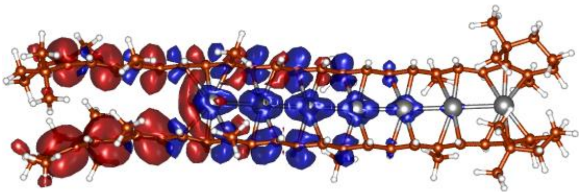

$[Pd_{10}(\beta\text{-carotene})_2]^{2+}$

D

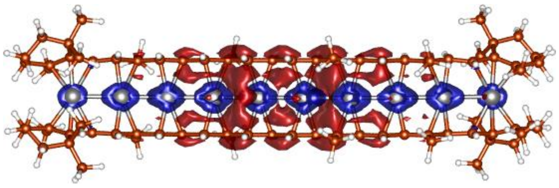

E

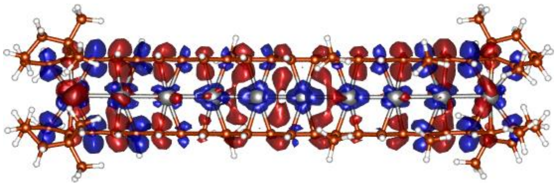

F

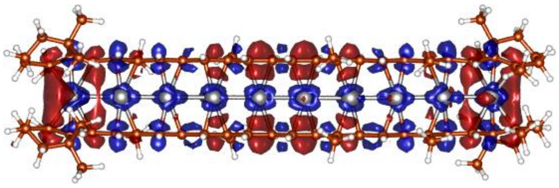

Supplementary Figure 4. Difference densities of the excited states A, B, C, D, E, and F (given in Supplementary Table 2) relative to the ground state for $[Pd_m(\beta\text{-carotene})_2]^{2+}$ (m = 5, 7, and 10). Blue (red) represents positive (negative) difference, *i.e.*, increase (decrease) in electron density.

0) −9.18 eV

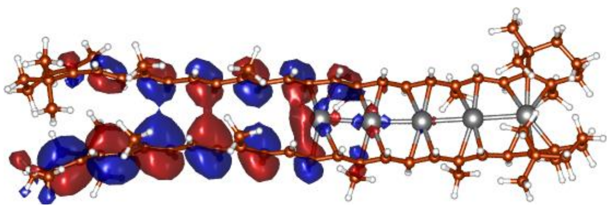

0´) −5.74 eV

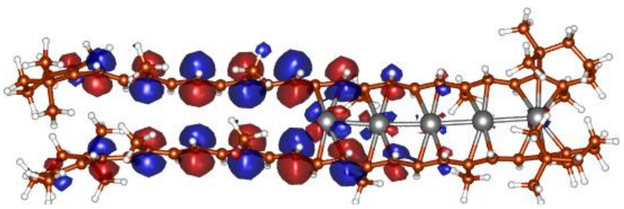

1) −9.42 eV

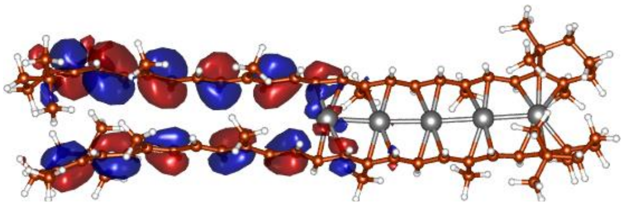

1´) −5.24 eV

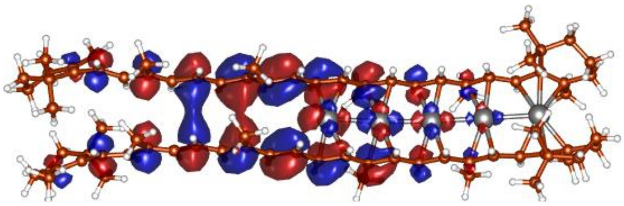

2) −10.45 eV

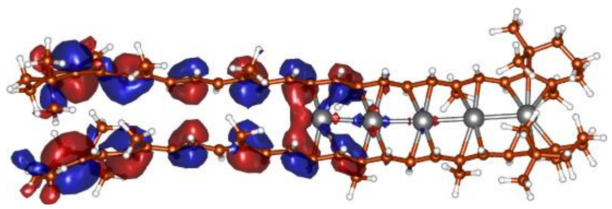

2´) −4.76 eV

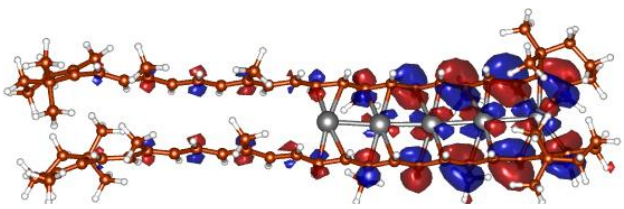

3) −10.70 eV

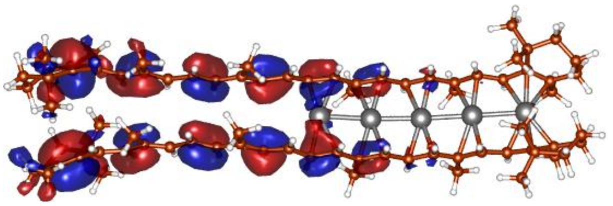

3´) −4.52 eV

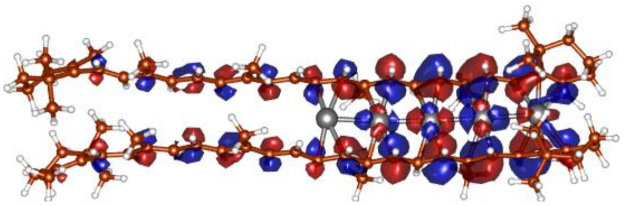

4) −10.77 eV

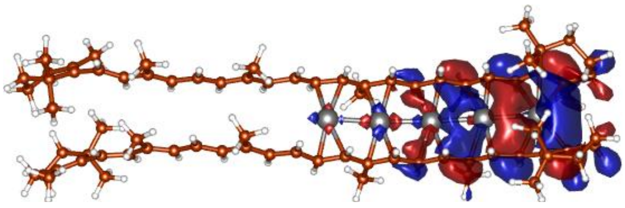

4´) −3.78 eV

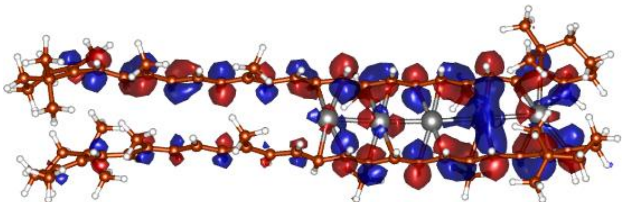

Supplementary Figure 5. Surface plots of HOMO − *i* and LUMO+*i* of [Pd_5(*β*-carotene)$_2$]$^{2+}$ for *i* = 0,..,4 along with their orbital energies (in eV). HOMO−*i* and LUMO+*i* are denoted by *i* and *i*´, respectively.

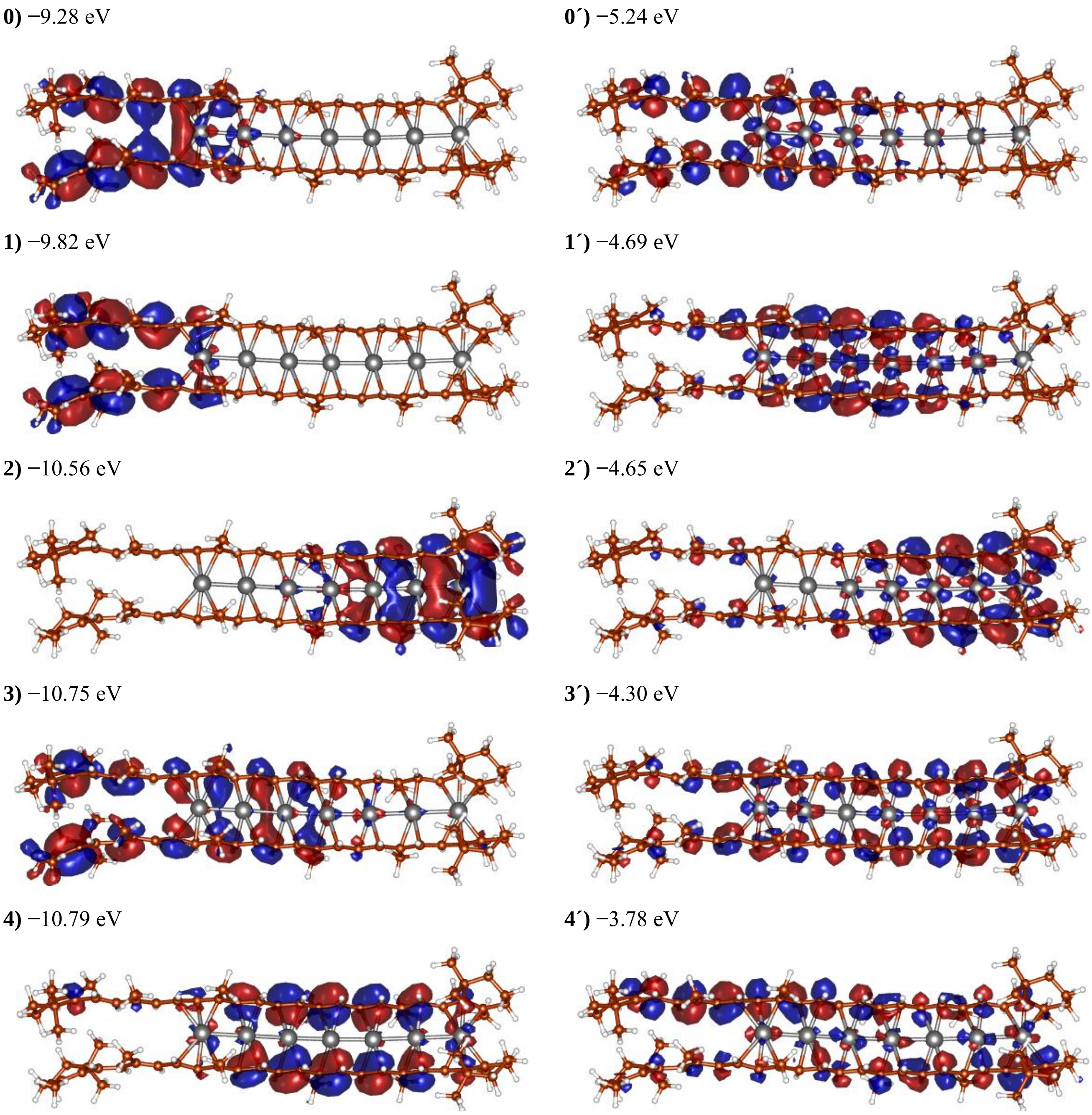

Supplementary Figure 6. Surface plots of HOMO − *i* and LUMO+*i* of [Pd_7(*β*-carotene)$_2$]$^{2+}$ for *i* = 0,..,4 along with their orbital energies (in eV). HOMO−*i* and LUMO+*i* are denoted by *i* and *i*´, respectively.

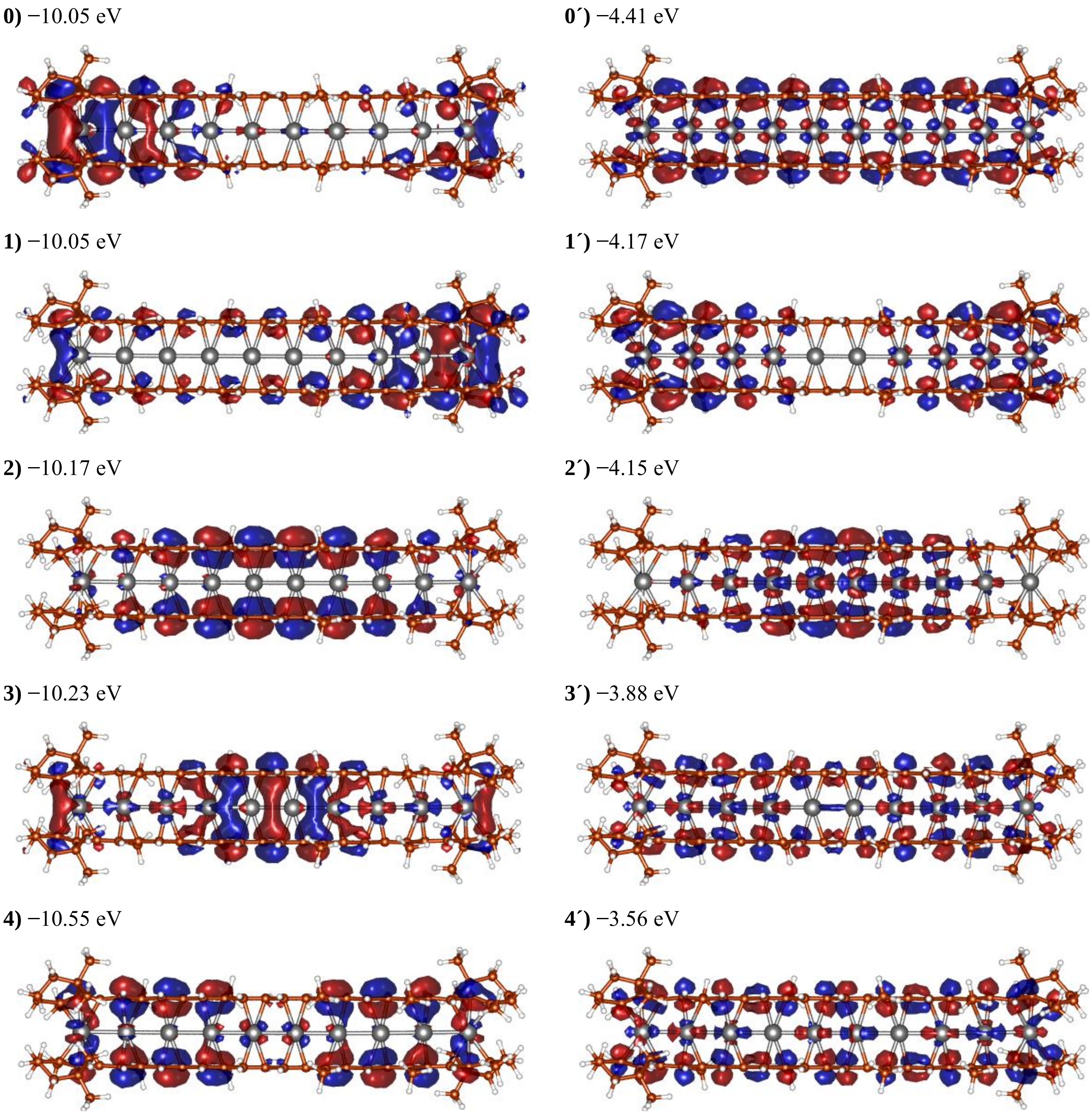

Supplementary Figure 7. Surface plots of HOMO − *i* and LUMO+*i* of $[Pd_{10}(\beta\text{-carotene})_2]^{2+}$ for *i* = 0,..,4 along with their orbital energies (in eV). HOMO−*i* and LUMO+*i* are denoted by *i* and *i*´, respectively.

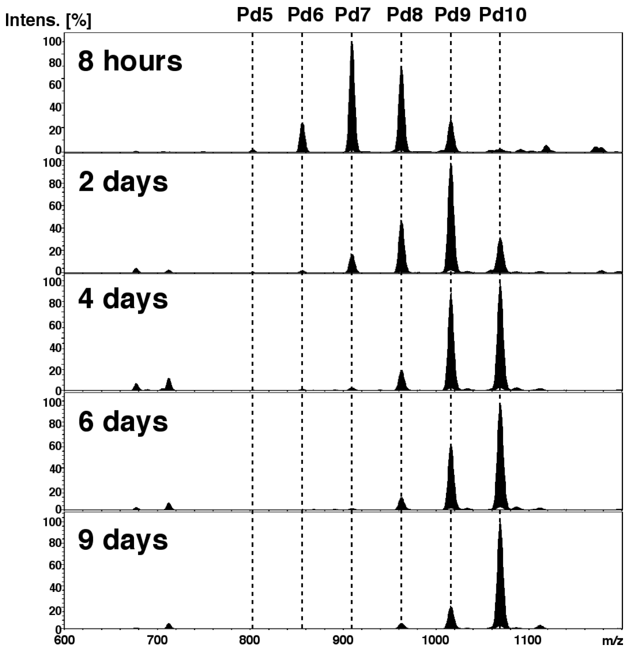

Supplementary Figure 8. ESI-MS monitoring of the formation of Pd_{10} complexes 1 with visible light irradiation (Xenon lamp, >385 nm) at 20 °C.

Supplementary Table 1. Selected intramolecular bond distances in X-ray determined structures.

a. 1-meso

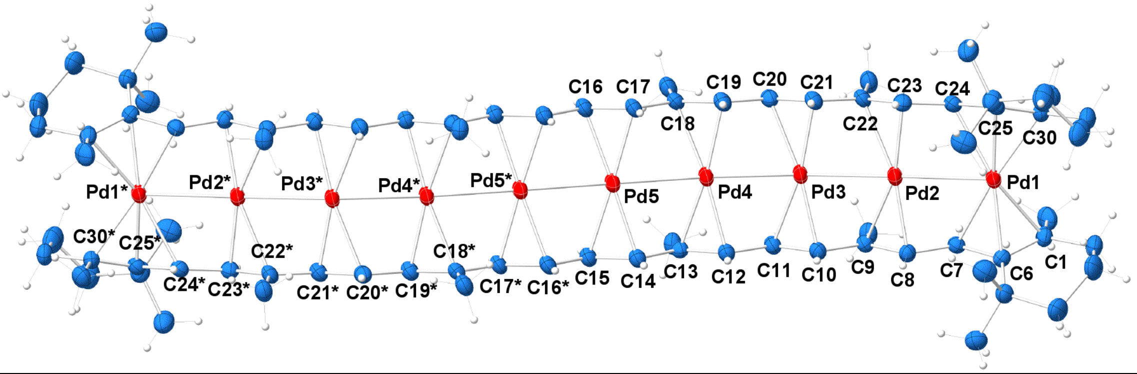

atom–atom	distance, Å	atom–atom	distance, Å	atom–atom	distance, Å
Pd1–Pd2	2.7172(6)	Pd3–C11	2.241(4)	C10–C11	1.412(5)
Pd2–Pd3	2.6115(6)	Pd3–C20	2.247(4)	C11–C12	1.448(5)
Pd3–Pd4	2.5901(6)	Pd3–C21	2.160(4)	C12–C13	1.422(5)
Pd4–Pd5	2.5882(7)	Pd4–C12	2.168(4)	C13–C14	1.449(5)
Pd5–Pd5*	2.5827(7)	Pd4–C13	2.223(4)	C14–C15	1.413(5)
		Pd4–C18	2.250(4)	C15–C16*	1.444(5)
Pd1–C1	2.244(4)	Pd4–C19	2.159(4)	C16–C17	1.405(5)
Pd1–C6	2.203(4)	Pd5–C14	2.195(4)	C17–C18	1.458(5)
Pd1–C7	2.335(4)	Pd5–C15	2.198(4)	C18–C19	1.419(5)
Pd1–C24	2.275(4)	Pd5–C16	2.212(4)	C19–C20	1.443(5)
Pd1–C25	2.201(4)	Pd5–C17	2.179(4)	C20–C21	1.411(5)
Pd1–C30	2.275(4)			C21–C22	1.445(5)
Pd2–C8	2.141(4)	C1–C6	1.421(5)	C22–C23	1.427(5)
Pd2–C9	2.308(4)	C6–C7	1.449(5)	C23–C24	1.435(5)
Pd2–C22	2.275(4)	C7–C8	1.435(5)	C24–C25	1.441(5)
Pd2–C23	2.149(4)	C8–C9	1.422(5)	C25–C30	1.416(5)
Pd3–C10	2.146(4)	C9–C10	1.450(5)		

b. 1-rac

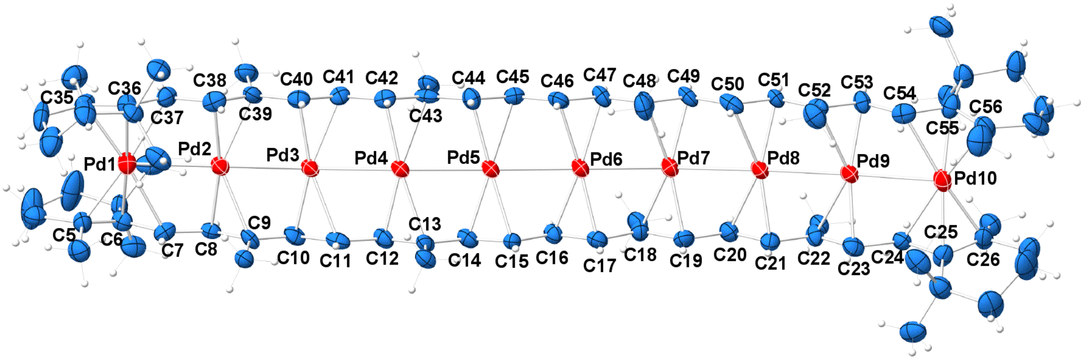

atom–atom	distance, Å	atom–atom	distance, Å	atom–atom	distance, Å
Pd1–Pd2	2.7068(11)	Pd6–C17	2.142(10)	C16–C17	1.371(14)
Pd2–Pd3	2.6170(11)	Pd6–C46	2.187(10)	C17–C18	1.449(13)
Pd3–Pd4	2.6155(10)	Pd6–C47	2.177(9)	C18–C19	1.439(14)
Pd4–Pd5	2.6105(11)	Pd7–C18	2.272(10)	C19–C20	1.426(14)
Pd5–Pd6	2.6037(11)	Pd7–C19	2.147(10)	C20–C21	1.379(14)
Pd6–Pd7	2.6010(11)	Pd7–C48	2.216(11)	C21–C22	1.414(13)
Pd7–Pd8	2.6142(11)	Pd7–C49	2.133(9)	C22–C23	1.411(14)
Pd8–Pd9	2.6125(11)	Pd8–C20	2.258(10)	C23–C24	1.465(14)
Pd9–Pd10	2.7111(11)	Pd8–C21	2.133(9)	C24–C25	1.422(14)
		Pd8–C50	2.257(11)	C25–C26	1.383(13)
Pd1–C5	2.209(10)	Pd8–C51	2.140(9)	C35–C36	1.380(15)
Pd1–C6	2.170(9)	Pd9–C22	2.333(9)	C36–C37	1.424(14)
Pd1–C7	2.382(10)	Pd9–C23	2.135(10)	C37–C38	1.425(15)
Pd1–C35	2.210(11)	Pd9–C52	2.313(9)	C38–C39	1.425(13)
Pd1–C36	2.142(10)	Pd9–C53	2.121(10)	C39–C40	1.434(14)
Pd1–C37	2.296(10)	Pd10–C24	2.306(9)	C40–C41	1.395(13)
Pd2–C8	2.150(8)	Pd10–C25	2.180(11)	C41–C42	1.424(14)
Pd2–C9	2.290(10)	Pd10–C26	2.200(12)	C42–C43	1.420(13)
Pd2–C38	2.137(10)	Pd10–C54	2.334(10)	C43–C44	1.476(15)
Pd2–C39	2.268(9)	Pd10–C55	2.169(11)	C44–C45	1.383(13)
Pd3–C10	2.136(9)	Pd10–C56	2.216(12)	C45–C46	1.479(14)
Pd3–C11	2.242(10)			C46–C47	1.363(13)
Pd3–C40	2.147(9)	C5–C6	1.420(13)	C47–C48	1.481(14)
Pd3–C41	2.255(9)	C6–C7	1.429(14)	C48–C49	1.376(13)
Pd4–C12	2.140(9)	C7–C8	1.419(14)	C49–C50	1.432(15)
Pd4–C13	2.250(9)	C8–C9	1.416(13)	C50–C51	1.409(13)
Pd4–C42	2.149(9)	C9–C10	1.421(13)	C51–C52	1.453(14)
Pd4–C43	2.260(8)	C10–C11	1.433(14)	C52–C53	1.421(13)
Pd5–C14	2.185(9)	C11–C12	1.436(14)	C53–C54	1.403(14)
Pd5–C15	2.177(9)	C12–C13	1.386(14)	C54–C55	1.435(15)

Pd5–C44	2.177(10)	C13–C14	1.419(13)	C55–C56	1.398(15)
Pd5–C45	2.218(9)	C14–C15	1.384(14)		
Pd6–C16	2.194(9)	C15–C16	1.421(12)		

c. 2-meso

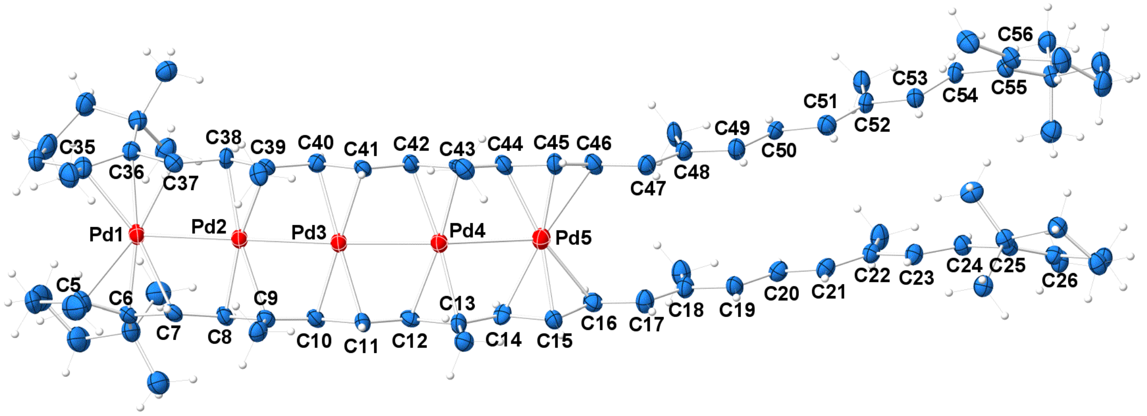

atom–atom	distance, Å	atom–atom	distance, Å	atom–atom	distance, Å
Pd1–Pd2	2.7501(7)	Pd5–C15	2.160(8)	C23–C24	1.347(9)
Pd2–Pd3	2.6605(7)	Pd5–C16	2.314(7)	C24–C25	1.479(10)
Pd3–Pd4	2.6546(7)	Pd5–C44	2.211(8)	C25–C26	1.374(9)
Pd4–Pd5	2.7252(8)	Pd5–C45	2.138(8)	C35–C36	1.420(11)
		Pd5–C46	2.420(8)	C36–C37	1.437(11)
Pd1–C5	2.288(9)			C37–C38	1.429(9)
Pd1–C6	2.235(9)	C5–C6	1.429(12)	C38–C39	1.423(11)
Pd1–C7	2.299(8)	C6–C7	1.465(11)	C39–C40	1.439(9)
Pd1–C35	2.298(8)	C7–C8	1.421(9)	C40–C41	1.424(10)
Pd1–C36	2.212(9)	C8–C9	1.425(11)	C41–C42	1.431(9)
Pd1–C37	2.267(8)	C9–C10	1.433(9)	C42–C43	1.426(10)
Pd2–C8	2.162(8)	C10–C11	1.437(10)	C43–C44	1.452(10)
Pd2–C9	2.248(8)	C11–C12	1.427(9)	C44–C45	1.406(10)
Pd2–C38	2.164(8)	C12–C13	1.426(10)	C45–C46	1.372(11)
Pd2–C39	2.260(8)	C13–C14	1.430(10)	C46–C47	1.437(10)
Pd3–C10	2.187(8)	C14–C15	1.419(10)	C47–C48	1.341(11)
Pd3–C11	2.192(8)	C15–C16	1.380(11)	C48–C49	1.457(10)
Pd3–C40	2.191(8)	C16–C17	1.435(10)	C49–C50	1.330(11)
Pd3–C41	2.216(8)	C17–C18	1.363(11)	C50–C51	1.451(10)
Pd4–C12	2.226(7)	C18–C19	1.440(10)	C51–C52	1.360(11)
Pd4–C13	2.186(8)	C19–C20	1.337(11)	C52–C53	1.466(10)
Pd4–C42	2.229(8)	C20–C21	1.443(11)	C53–C54	1.344(11)
Pd4–C43	2.210(8)	C21–C22	1.348(10)	C54–C55	1.464(10)
Pd5–C14	2.296(8)	C22–C23	1.449(11)	C55–C56	1.353(12)

d. 3-meso

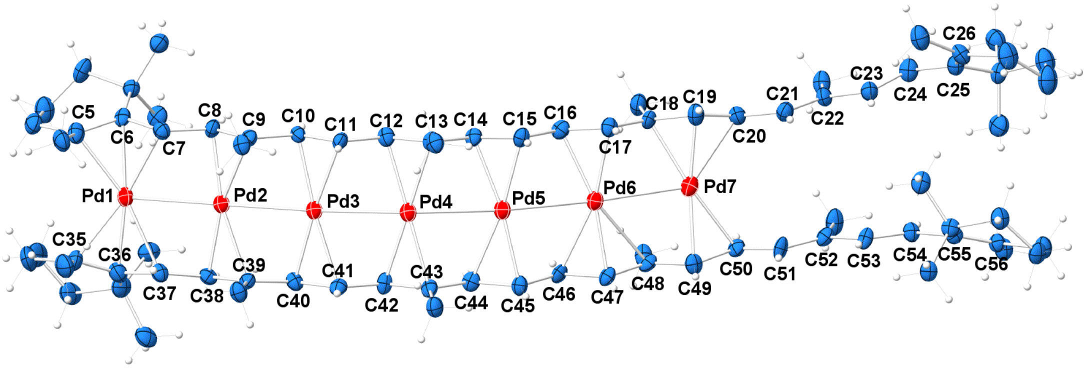

atom–atom	distance, Å	atom–atom	distance, Å	atom–atom	distance, Å
Pd1–Pd2	2.7359(9)	Pd5–C44	2.267(10)	C20–C21	1.445(12)
Pd2–Pd3	2.6416(8)	Pd5–C45	2.172(9)	C21–C22	1.385(14)
Pd3–Pd4	2.6285(8)	Pd6–C16	2.209(9)	C22–C23	1.457(13)
Pd4–Pd5	2.6437(9)	Pd6–C17	2.165(10)	C23–C24	1.353(14)
Pd5–Pd6	2.6465(9)	Pd6–C46	2.343(10)	C24–C25	1.476(14)
Pd6–Pd7	2.7074(10)	Pd6–C47	2.144(10)	C25–C26	1.341(16)
		Pd7–C18	2.186(9)	C35–C36	1.393(14)
Pd1–C5	2.275(10)	Pd7–C19	2.086(11)	C36–C37	1.452(14)
Pd1–C6	2.220(10)	Pd7–C20	2.386(10)	C37–C38	1.403(11)
Pd1–C7	2.267(10)	Pd7–C49	2.164(10)	C38–C39	1.428(13)
Pd1–C35	2.266(12)	Pd7–C50	2.240(8)	C39–C40	1.424(11)
Pd1–C36	2.210(11)			C40–C41	1.434(13)
Pd1–C37	2.337(10)	C5–C6	1.417(13)	C41–C42	1.445(12)
Pd2–C8	2.122(10)	C6–C7	1.440(13)	C42–C43	1.401(12)
Pd2–C9	2.240(10)	C7–C8	1.445(11)	C43–C44	1.437(13)
Pd2–C38	2.127(10)	C8–C9	1.388(13)	C44–C45	1.432(12)
Pd2–C39	2.290(10)	C9–C10	1.462(11)	C45–C46	1.430(13)
Pd3–C10	2.165(9)	C10–C11	1.406(12)	C46–C47	1.404(11)
Pd3–C11	2.218(10)	C11–C12	1.435(12)	C47–C48	1.433(13)
Pd3–C40	2.151(9)	C12–C13	1.420(12)	C48–C49	1.435(12)
Pd3–C41	2.247(10)	C13–C14	1.451(12)	C49–C50	1.398(13)
Pd4–C12	2.196(10)	C14–C15	1.409(11)	C50–C51	1.449(12)
Pd4–C13	2.213(10)	C15–C16	1.428(13)	C51–C52	1.369(13)
Pd4–C42	2.184(10)	C16–C17	1.436(12)	C52–C53	1.470(13)
Pd4–C43	2.202(10)	C17–C18	1.444(13)	C53–C54	1.340(12)
Pd5–C14	2.187(10)	C18–C19	1.436(12)	C54–C55	1.484(13)
Pd5–C15	2.169(10)	C19–C20	1.398(13)	C55–C56	1.362(11)

e. 4-meso

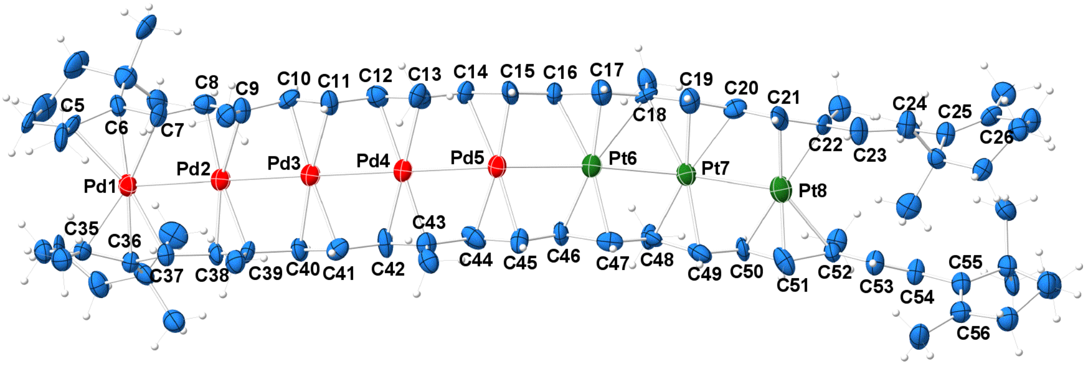

atom–atom	distance, Å	atom–atom	distance, Å	atom–atom	distance, Å
Pd1–Pd2	2.7305(17)	Pd5–C45	2.18(2)	C18–C19	1.43(2)
Pd2–Pd3	2.6348(17)	Pt6–C16	2.279(18)	C19–C20	1.41(3)
Pd3–Pd4	2.6290(16)	Pt6–C17	2.13(2)	C20–C21	1.47(3)
Pd4–Pd5	2.6381(16)	Pt6–C46	2.190(17)	C21–C22	1.32(3)
Pd5–Pt6	2.6569(13)	Pt6–C47	2.19(2)	C22–C23	1.46(2)
Pt6–Pt7	2.6612(9)	Pt7–C18	2.479(18)	C23–C24	1.39(3)
Pt7–Pt8	2.6864(10)	Pt7–C19	2.15(2)	C24–C25	1.46(3)
		Pt7–C20	2.292(16)	C25–C26	1.40(2)
Pd1–C5	2.306(19)	Pt7–C48	2.177(18)	C35–C36	1.42(2)
Pd1–C6	2.240(18)	Pt7–C49	2.24(2)	C36–C37	1.45(3)
Pd1–C7	2.357(16)	Pt8–C21	2.13(2)	C37–C38	1.41(2)
Pd1–C35	2.257(16)	Pt8–C22	2.208(15)	C38–C39	1.37(2)
Pd1–C36	2.258(19)	Pt8–C50	2.162(17)	C39–C40	1.50(2)
Pd1–C37	2.253(17)	Pt8–C51	2.03(2)	C40–C41	1.40(3)
Pd2–C8	2.16(2)	Pt8–C52	2.37(2)	C41–C42	1.43(2)
Pd2–C9	2.283(18)			C42–C43	1.40(2)
Pd2–C38	2.145(18)	C5–C6	1.47(2)	C43–C44	1.50(2)
Pd2–C39	2.215(17)	C6–C7	1.54(2)	C44–C45	1.41(3)
Pd3–C10	2.213(19)	C7–C8	1.35(2)	C45–C46	1.45(3)
Pd3–C11	2.243(19)	C8–C9	1.50(2)	C46–C47	1.50(2)
Pd3–C40	2.214(18)	C9–C10	1.43(2)	C47–C48	1.45(2)
Pd3–C41	2.228(19)	C10–C11	1.45(3)	C48–C49	1.56(3)
Pd4–C12	2.20(2)	C11–C12	1.45(2)	C49–C50	1.40(3)
Pd4–C13	2.22(2)	C12–C13	1.45(3)	C50–C51	1.37(3)
Pd4–C42	2.181(18)	C13–C14	1.45(2)	C51–C52	1.47(3)
Pd4–C43	2.166(19)	C14–C15	1.38(2)	C52–C53	1.45(3)
Pd5–C14	2.257(18)	C15–C16	1.45(2)	C53–C54	1.29(3)
Pd5–C15	2.151(18)	C16–C17	1.43(3)	C54–C55	1.52(3)
Pd5–C44	2.19(2)	C17–C18	1.47(3)	C55–C56	1.31(3)

Supplementary Table 2. Low-lying vertical excitation states that have the oscillator strength, denoted *f*, larger than 0.53 for $[Pd_m(\beta\text{-carotene})_2]^{2+}$ (*m* = 5, 7, and 10). Besides the energies of the excited states (in eV), their characters are presented using relevant singly-excited configurations from HOMO−*i* (denoted *i*) to LUMO+*i* (denoted *i'*); their percentages of total weight are shown in parentheses.

	state label	excitation energy (eV)	oscillator strength f [a)]	main configurations: [b)] *i*:HOMO−*i* *i´*:LUMO+*i*			
$[Pd_5(\beta\text{-carotene})_2]^{2+}$	**A**	2.77	6.967	0→1´(45%),	1→0´(20%),	1→1´(17%)	
	**B**	3.45	0.939	3→0´(45%),	0→3´(15%),	2→1´(14%)	
$[Pd_7(\beta\text{-carotene})_2]^{2+}$	**C**	3.21	4.279	0→1´(58%),	1→2´(15%),	0→3´(12%)	
$[Pd_{10}(\beta\text{-carotene})_2]^{2+}$	**D**	3.77	0.595	1→2´(22%),	2→0´(20%),	3→2´(12%),	0→3´(11%)
	**E**	3.91	3.856	3→2´(20%),	2→0´(20%),	4→1´(19%)	
	**F**	4.00	3.054	2→0´(24%),	0→3´(19%),	1→4´(11%)	

a) States with *f* larger than 0.53 are shown.

b) Excited configurations with a weight less than 10% are discarded.

Supplementary Table 3. Cartesian coordinates (in Å) of the optimized geometry of [Pd_5(*β*-carotene)$_2$].

	x	*y*	*z*
Pd	9.907064	0.158485	-0.199127
Pd	7.132613	-0.011139	-0.362574
Pd	4.487052	-0.156983	-0.488304
Pd	1.861907	-0.174287	-0.300969
Pd	-0.781022	-0.039810	0.186826
C	12.695680	-1.393093	0.737057
C	12.627160	-0.990069	2.203319
C	12.550801	2.162293	-0.522778
C	12.431736	2.806597	0.851597
C	11.694333	-1.955296	2.923998
C	11.493618	-2.133442	-1.335243
C	11.344214	-1.612698	0.088935
C	11.410235	1.261385	-2.568420
C	11.223374	1.790909	-1.151452
C	11.404037	3.927961	0.771417
C	10.226094	-1.862782	2.431815
C	10.171945	-1.781898	0.878294
C	10.004189	2.345265	-0.668518
C	9.533238	-3.176782	2.873685
C	9.972003	3.422448	0.455054
C	9.564406	-0.651688	3.127639
C	8.921237	-1.921737	0.156121
C	9.345013	2.900663	1.768537
C	9.181725	4.651106	-0.061738
C	8.787240	1.873542	-1.300943
C	7.575395	-1.878762	0.660865
C	7.421444	2.076621	-0.901082
C	6.423966	-2.923590	-1.402286
C	6.391182	-2.188510	-0.076432
C	6.319273	1.311134	-3.111996
C	6.262770	1.703606	-1.648510
C	5.106306	-1.983850	0.581121
C	4.961813	1.924551	-1.030112
C	3.830435	-2.262650	0.030069
C	3.697612	1.647756	-1.607741
C	2.583364	-2.017596	0.727750
C	2.444091	1.887526	-0.922387
C	1.269512	-2.284520	0.245177
C	1.018296	-3.076285	-1.023607
C	1.132331	1.651516	-1.425892
C	0.881870	1.272122	-2.871361
C	0.131853	-1.912539	1.072559
C	0.003159	1.934650	-0.557108
C	-1.232732	-2.138225	0.734914
C	-1.377949	1.804663	-0.888562
C	-2.335098	-1.695814	1.508963
C	-2.404995	2.011524	0.064194
C	-3.663226	-1.822424	1.061379
C	-3.776857	1.807948	-0.176082
C	-4.859136	-0.884867	3.111243
C	-4.840894	-1.445182	1.711566
C	-4.443162	2.496758	2.149388
C	-4.794739	2.010197	0.760789
C	-6.067689	-1.646671	1.019263
C	-6.144185	1.790268	0.361462

C	-7.341331	-1.386488	1.490039
C	-7.279769	2.011207	1.118390
C	-8.500402	-1.629823	0.708517
C	-8.604667	1.864266	0.626625
C	-9.829364	-1.500534	1.078144
C	-9.791047	2.673772	2.724372
C	-9.773745	2.180252	1.298433
C	-10.265100	-1.058554	2.451454
C	-10.827017	-1.831690	0.089234
C	-11.028734	2.055072	0.592596
C	-12.175043	-1.891439	0.309289
C	-12.247449	2.414025	1.095218
C	-12.352336	-0.509529	-2.359610
C	-12.226444	-2.999042	-2.812307
C	-12.672008	3.187799	-1.807185
C	-13.052847	-1.872105	-2.145986
C	-13.258034	-2.156974	-0.634431
C	-13.544826	2.321504	0.428459
C	-13.715685	2.572749	-0.905284
C	-14.455022	-2.625777	-0.148134
C	-14.733341	-2.970831	1.294950
C	-14.504820	0.508924	1.895475
C	-14.421846	-1.766267	-2.866276
C	-14.805916	2.915176	2.561735
C	-14.723199	1.941103	1.362251
C	-15.022760	2.298383	-1.613757
C	-15.374258	-2.903969	-2.519921
C	-15.663069	-2.876488	-1.022618
C	-16.076000	2.016854	0.615352
C	-15.995157	1.451267	-0.798651
H	13.635542	-1.010567	2.649728
H	13.266884	-2.340024	0.643705
H	13.260952	-0.655431	0.140986
H	13.196459	1.266734	-0.493219
H	13.412223	3.196007	1.174209
H	13.061166	2.864239	-1.214926
H	12.283851	-1.577166	-1.864724
H	12.280906	0.587212	-2.611150
H	12.260345	0.047727	2.294800
H	12.067172	-2.984941	2.765313
H	12.127254	2.051802	1.598235
H	11.705716	-1.785998	4.014915
H	11.823652	-3.190748	-1.289120
H	11.632687	2.115981	-3.238093
H	11.722604	4.635588	-0.017136
H	10.556021	0.716386	-2.996547
H	10.591664	-2.094202	-1.963484
H	11.367198	4.507019	1.710794
H	9.932563	-4.041073	2.313297
H	10.177413	0.253984	3.003208
H	9.733919	-3.349827	3.945806
H	10.016048	2.179835	2.260025
H	9.469998	-0.848491	4.210159
H	9.009769	-2.302722	-0.862123
H	9.552652	4.973961	-1.051054
H	8.907849	1.482787	-2.311907
H	9.181245	3.741531	2.465758
H	8.440190	-3.165088	2.741364
H	9.321572	5.492818	0.639519

H	8.563130	-0.412355	2.737573
H	8.380923	2.388956	1.622759
H	8.099094	4.473168	-0.148794
H	7.404379	-2.863521	-1.896155
H	7.428702	-1.642394	1.715916
H	7.325156	1.002267	-3.431179
H	7.228970	2.616165	0.027505
H	6.196352	-3.994150	-1.236464
H	5.679076	-2.527179	-2.111283
H	5.635420	0.476561	-3.337433
H	6.018565	2.173160	-3.737948
H	5.148192	-1.683335	1.636191
H	4.972135	2.454428	-0.068596
H	3.776586	-2.790451	-0.925462
H	3.652508	1.346612	-2.657763
H	2.676158	-1.690952	1.772089
H	2.521870	2.411322	0.039564
H	1.919941	-3.174848	-1.644862
H	1.755682	0.792231	-3.337092
H	0.672241	-4.095828	-0.767122
H	0.240645	-2.606546	-1.648166
H	0.358130	-1.582372	2.094959
H	0.037610	0.570273	-2.966230
H	0.637320	2.176491	-3.461148
H	0.241258	2.445797	0.385245
H	-1.465257	-2.686286	-0.185128
H	-1.672737	1.542550	-1.911172
H	-2.131573	-1.283788	2.504338
H	-2.098745	2.405613	1.040335
H	-3.783257	-2.249595	0.057412
H	-3.857657	-0.624439	3.485165
H	-5.296056	-1.619107	3.813458
H	-4.005411	3.511153	2.110742
H	-4.063401	1.451672	-1.173400
H	-3.697028	1.834268	2.621627
H	-5.484062	0.022731	3.163450
H	-5.309723	2.542732	2.823566
H	-5.989356	-2.054413	0.003257
H	-6.291810	1.438113	-0.667303
H	-7.463119	-0.998112	2.505897
H	-7.157925	2.367177	2.144874
H	-8.311954	-1.983616	-0.313527
H	-8.704510	1.505048	-0.404927
H	-10.176185	3.708091	2.778375
H	-9.432546	-0.681259	3.063491
H	-8.800696	2.661511	3.201332
H	-10.732285	-1.898311	2.999492
H	-10.461401	2.049307	3.341686
H	-10.430366	-2.078704	-0.897328
H	-11.020902	-0.257687	2.376375
H	-10.976188	1.632846	-0.415969
H	-11.283700	-0.519209	-2.095902
H	-11.224189	-3.105889	-2.368029
H	-12.200136	2.427672	-2.459666
H	-12.278721	2.751526	2.135418
H	-12.495546	-1.708626	1.337297
H	-11.874064	3.711114	-1.258485
H	-12.425984	-0.215435	-3.422154
H	-12.092287	-2.784007	-3.888478

H	-12.839125	0.269560	-1.749246
H	-13.159305	3.914100	-2.484736
H	-12.726807	-3.977254	-2.718886
H	-13.532232	0.418149	2.413844
H	-13.979615	2.782422	3.281960
H	-14.758788	-4.072397	1.410931
H	-14.007086	-2.583951	2.023460
H	-14.509280	-0.222267	1.072381
H	-14.242377	-1.721149	-3.955577
H	-14.807990	3.967129	2.224331
H	-14.792523	1.814048	-2.583551
H	-14.897272	-0.806163	-2.587585
H	-14.930580	-3.876245	-2.799830
H	-15.297830	0.233152	2.614111
H	-15.743988	2.735846	3.117322
H	-15.734944	-2.603832	1.585408
H	-15.492975	3.267494	-1.881517
H	-15.644128	0.403479	-0.768882
H	-16.159185	-3.810137	-0.696782
H	-16.394472	-2.071117	-0.795275
H	-16.397018	3.074377	0.553445
H	-16.314510	-2.813821	-3.090705
H	-16.842435	1.491489	1.213181
H	-16.989656	1.441118	-1.277153

Supplementary Table 4. Cartesian coordinates (in Å) of the optimized geometry of [Pd_7(*β*-carotene)$_2$].

	x	*y*	*z*
Pd	10.406195	0.128887	-0.183925
Pd	7.639112	0.017250	-0.394590
Pd	5.000214	-0.072729	-0.556675
Pd	2.384299	-0.066244	-0.419878
Pd	-0.211820	0.018005	-0.033223
Pd	-2.770921	0.161671	0.533979
Pd	-5.447770	0.297219	0.979010
C	13.141094	-1.557763	0.656588
C	13.045143	-1.296762	2.153413
C	13.075267	2.104363	-0.273953
C	12.949131	2.613761	1.155250
C	11.971180	-2.062769	-1.505425
C	12.078644	-2.307444	2.758014
C	11.949320	1.430364	-2.411633
C	11.801032	-1.685182	-0.038413
C	11.750334	1.820073	-0.952028
C	11.941947	3.756124	1.173213
C	10.608018	-1.915863	0.703435
C	10.624151	-2.145591	2.243309
C	10.533522	2.346950	-0.430696
C	9.901571	-3.483067	2.545132
C	10.506220	3.308930	0.793552
C	9.960598	-0.994537	3.032399
C	9.371999	-1.967010	-0.055313
C	9.855151	2.675449	2.044711
C	9.314551	1.963861	-1.117455
C	9.742393	4.594916	0.388503
C	8.015986	-1.959520	0.423762
C	7.948382	2.145318	-0.705435
C	6.889268	-2.747509	-1.764319
C	6.842272	-2.168334	-0.364053
C	6.843713	1.649712	-2.990764
C	6.786933	1.876266	-1.492700
C	5.547397	-2.024240	0.290938
C	5.484841	2.048458	-0.859335
C	4.275736	-2.214942	-0.308148
C	4.218569	1.860133	-1.468961
C	3.022411	-2.032743	0.398687
C	2.962260	2.048699	-0.768436
C	1.707260	-2.214753	-0.120393
C	1.456939	-2.855369	-1.471953
C	1.647179	1.903452	-1.298335
C	1.389443	1.731913	-2.782520
C	0.568783	-1.944865	0.751653
C	0.510411	2.113041	-0.407940
C	-0.798739	-2.078190	0.406022
C	-0.860479	2.023146	-0.757053
C	-1.905746	-1.807383	1.302761
C	-1.948795	2.249012	0.173786
C	-3.261748	-1.941570	0.914753
C	-3.322061	2.173984	-0.174320
C	-4.286953	-1.542850	3.230071
C	-4.158248	2.948930	2.107661
C	-4.436787	-1.698223	1.730192
C	-4.439683	2.381977	0.726886

C	-5.711139	-1.874693	1.095055
C	-5.775605	2.305348	0.191016
C	-6.984727	-1.519450	1.589201
C	-6.951901	2.279766	0.966957
C	-8.149484	-1.676878	0.776016
C	-8.271962	2.138448	0.442190
C	-9.474111	-1.497513	1.118725
C	-9.467056	2.610458	2.630629
C	-9.445900	2.281051	1.157051
C	-9.922603	-1.059618	2.488993
C	-10.463947	-1.788851	0.103651
C	-10.706992	2.162975	0.455989
C	-11.813094	-1.840848	0.299546
C	-11.928172	2.435943	1.002382
C	-11.956950	-0.422661	-2.354317
C	-11.815629	-2.904083	-2.841907
C	-12.446207	3.275355	-1.876774
C	-12.655616	-1.790056	-2.170410
C	-12.882366	-2.099463	-0.666790
C	-13.241344	2.348488	0.364797
C	-13.452411	2.622432	-0.958463
C	-14.080728	-2.587763	-0.208849
C	-14.379005	-2.958782	1.224336
C	-14.147653	0.520037	1.856807
C	-14.466148	2.924506	2.521563
C	-14.014210	-1.678438	-2.908847
C	-14.393614	1.947113	1.323527
C	-14.768271	2.324209	-1.640773
C	-14.963794	-2.829194	-2.599154
C	-15.273756	-2.835638	-1.105662
C	-15.763882	2.002158	0.606534
C	-15.701536	1.446955	-0.812201
H	14.041792	-1.381080	2.618445
H	13.697941	-2.502253	0.483076
H	13.732384	-0.776107	0.147860
H	13.705411	1.198573	-0.323659
H	13.931923	2.952169	1.524785
H	13.607936	2.861693	-0.886347
H	12.814338	-1.505811	-1.944863
H	12.787488	0.720986	-2.506301
H	12.696081	-0.264831	2.336280
H	12.439936	-3.324204	2.512500
H	12.623491	1.795966	1.822068
H	12.227986	-3.139289	-1.568664
H	12.065694	-2.240587	3.859941
H	12.230456	2.337220	-2.983508
H	12.283295	4.531229	0.461062
H	11.080464	0.978337	-2.912210
H	11.097589	-1.889221	-2.150976
H	11.903589	4.241978	2.164100
H	10.297567	-4.297637	1.912671
H	10.574121	-0.082339	2.985614
H	10.076750	-3.761964	3.599377
H	10.508995	1.902812	2.477732
H	9.858948	-1.279712	4.094479
H	9.478967	-2.241310	-1.105719
H	10.137882	5.008014	-0.556735
H	9.434326	1.679267	-2.163553
H	9.691374	3.450513	2.814485

H	8.811967	-3.438343	2.394415
H	9.879896	5.360827	1.172338
H	8.961657	-0.724885	2.656831
H	8.887683	2.191944	1.839970
H	8.659243	4.443331	0.263114
H	7.876433	-2.642226	-2.237071
H	7.849494	-1.838572	1.495426
H	7.854232	1.398428	-3.343940
H	7.757879	2.579820	0.277087
H	6.523335	2.569987	-3.515873
H	6.650049	-3.827631	-1.724073
H	6.156579	-2.264954	-2.431479
H	6.174790	0.832539	-3.306979
H	5.573908	-1.852935	1.375249
H	5.498081	2.470324	0.154471
H	4.230210	-2.626605	-1.319968
H	4.176483	1.673961	-2.545389
H	3.109500	-1.831992	1.474982
H	3.044363	2.447784	0.251549
H	2.340689	-2.819959	-2.125492
H	2.279021	1.382770	-3.326619
H	1.082003	2.699933	-3.222832
H	1.178159	-3.918301	-1.337530
H	0.635555	-2.357761	-2.013072
H	0.807078	-1.727617	1.801006
H	0.750375	2.486968	0.596006
H	0.583896	1.004334	-2.974536
H	-1.062835	-2.515204	-0.563784
H	-1.139512	1.882964	-1.807853
H	-1.657578	-1.610288	2.352128
H	-1.668667	2.617400	1.167054
H	-3.510174	-0.804708	3.489388
H	-3.452969	-2.348212	-0.087356
H	-3.571091	2.031556	-1.234403
H	-3.398959	2.360202	2.648732
H	-3.773554	3.982059	2.007641
H	-3.990456	-2.513999	3.671106
H	-5.221661	-1.226772	3.716544
H	-5.054081	2.990217	2.742823
H	-5.685497	-2.271452	0.071186
H	-5.879249	2.219216	-0.898896
H	-7.105544	-1.267967	2.647474
H	-6.861761	2.514622	2.030432
H	-7.952178	-1.995042	-0.255971
H	-8.354404	1.914397	-0.628789
H	-9.833384	3.640310	2.795751
H	-9.094310	-0.688515	3.112089
H	-8.480906	2.529430	3.110556
H	-10.405847	-1.894921	3.029839
H	-10.151477	1.936547	3.173728
H	-10.052535	-2.023602	-0.880055
H	-10.670656	-0.251922	2.406045
H	-10.648895	1.828278	-0.583851
H	-10.899094	-0.426603	-2.050094
H	-10.816123	-3.007813	-2.390845
H	-11.952747	2.534548	-2.535737
H	-11.947173	2.697072	2.064310
H	-12.151512	-1.666937	1.323360
H	-11.662684	3.830975	-1.338432

H	-11.994470	-0.126057	-3.418214
H	-11.674619	-2.677263	-3.914845
H	-12.472233	0.350748	-1.760365
H	-12.971074	3.981329	-2.547498
H	-12.309427	-3.886830	-2.762184
H	-13.165137	0.440994	2.357343
H	-13.620558	2.811944	3.222732
H	-14.408465	-4.062129	1.320530
H	-13.661166	-2.586806	1.968863
H	-13.819753	-1.610484	-3.994497
H	-14.159558	-0.213089	1.035904
H	-14.496710	3.974709	2.180237
H	-14.545131	1.851607	-2.618186
H	-14.499564	-0.727155	-2.617632
H	-14.509554	-3.792281	-2.893547
H	-14.923880	0.237395	2.590830
H	-15.387032	2.728903	3.100104
H	-15.267994	3.282770	-1.892400
H	-15.383520	-2.594550	1.508792
H	-15.323019	0.408403	-0.796663
H	-15.761342	-3.782918	-0.807182
H	-16.105928	3.053687	0.557432
H	-16.019892	-2.045733	-0.872836
H	-15.896473	-2.733242	-3.181438
H	-16.507649	1.459422	1.217257
H	-16.705678	1.414967	-1.269147

Supplementary Table 5. Cartesian coordinates (in Å) of the optimized geometry of [Pd_{10}(*β*-carotene)$_2$].

	x	*y*	*z*
Pd	11.878285	-0.023106	0.172012
Pd	9.128875	-0.059865	0.412255
Pd	6.497909	-0.094511	0.597388
Pd	3.878308	-0.098624	0.524506
Pd	1.280899	-0.093546	0.217881
Pd	-1.286754	-0.080726	-0.253561
Pd	-3.884199	-0.070035	-0.558108
Pd	-6.504472	-0.060865	-0.622642
Pd	-9.134271	-0.037888	-0.424115
Pd	-11.881737	-0.012526	-0.160296
C	14.569545	1.892542	0.141170
C	14.490743	2.250723	-1.336417
C	14.529577	-1.823501	-0.602885
C	14.359580	-1.695995	-2.110142
C	13.516307	3.411091	-1.497117
C	13.462152	-2.106106	1.650644
C	13.362147	-2.750343	-2.571466
C	13.370389	1.458775	2.300181
C	13.219430	1.705009	0.803522
C	13.222620	-1.860149	0.165542
C	12.059274	3.040347	-1.113363
C	12.028666	2.204732	0.199532
C	11.991320	-2.154139	-0.493124
C	11.935267	-2.535094	-2.002349
C	11.421320	2.304900	-2.313304
C	11.320209	4.377235	-0.855197
C	11.203178	-3.893967	-2.140214
C	11.229280	-1.466920	-2.867811
C	10.780090	1.933265	0.889611
C	10.788562	-2.106943	0.316856
C	9.427087	2.090308	0.421856
C	9.410380	-2.150544	-0.096380
C	8.385214	-2.536435	2.241877
C	8.268270	1.963752	2.725651
C	8.239548	1.958046	1.209193
C	8.270459	-2.212224	0.764808
C	6.947006	2.044357	0.532740
C	6.944002	-2.183919	0.152904
C	5.667411	1.975874	1.147325
C	5.697914	-2.232313	0.833290
C	4.405261	2.038854	0.427259
C	4.404636	-2.207432	0.169071
C	3.090939	2.008627	0.988515
C	3.117760	-2.246299	0.788033
C	2.861085	2.151939	2.481166
C	2.958248	-2.526955	2.270047
C	1.930685	2.032827	0.096846
C	1.921287	-2.204573	-0.052960
C	0.562085	2.025194	0.483457
C	0.569658	-2.220430	0.386597
C	-0.558737	2.043468	-0.443169
C	-0.583627	-2.202939	-0.498714
C	-1.926763	2.041810	-0.054237
C	-1.935530	-2.198521	-0.060020
C	-2.867875	2.252205	-2.429059

C	-2.964162	-2.432237	-2.395975
C	-3.090168	2.052742	-0.941794
C	-3.129884	-2.206821	-0.905169
C	-4.401856	2.063293	-0.373754
C	-4.418343	-2.190737	-0.288768
C	-5.667574	2.029946	-1.089275
C	-5.710395	-2.184912	-0.956209
C	-6.942629	2.074450	-0.463089
C	-6.957632	-2.168383	-0.276439
C	-8.241553	2.017452	-1.129841
C	-8.284076	-2.165673	-0.890104
C	-8.398770	-2.410455	-2.382330
C	-8.289741	2.093609	-2.643682
C	-9.419506	2.111645	-0.323182
C	-9.423989	-2.151180	-0.026591
C	-10.779265	1.980520	-0.778064
C	-10.802971	-2.083248	-0.435319
C	-11.354096	2.163235	2.452029
C	-11.281833	4.319015	1.119906
C	-11.220788	-4.024966	1.896678
C	-11.231637	-1.651961	2.787309
C	-12.016399	2.968088	1.311194
C	-12.014379	2.213982	-0.050781
C	-12.004012	-2.177945	0.373013
C	-11.945317	-2.655888	1.853523
C	-13.219749	1.757013	-0.660466
C	-13.236377	-1.839365	-0.262117
C	-13.482859	-1.998792	-1.757826
C	-13.464833	3.316545	1.745862
C	-13.403740	1.605831	-2.165907
C	-13.371376	-2.901545	2.411955
C	-14.444372	2.168711	1.535474
C	-14.555883	1.905383	0.040337
C	-14.363935	-1.813284	2.024794
C	-14.540124	-1.841684	0.512963
H	15.490285	2.521559	-1.716506
H	15.331098	-1.826826	-2.616021
H	15.175014	0.983279	0.304784
H	15.103882	2.699667	0.684766
H	15.156402	-1.008104	-0.200705
H	15.083976	-2.758082	-0.374658
H	14.153698	1.376076	-1.920445
H	14.320636	-1.509978	2.001296
H	14.181000	0.735990	2.488359
H	14.002599	-0.682073	-2.364500
H	13.861538	4.246590	-0.858449
H	13.728905	-3.172069	1.798626
H	13.735143	-3.742621	-2.252872
H	13.515276	3.793675	-2.533066
H	13.667351	2.408661	2.788692
H	13.293589	-2.783830	-3.672942
H	12.616292	-1.878175	2.316022
H	12.474995	1.084134	2.817841
H	12.067868	1.482649	-2.657380
H	11.630031	-4.644944	-1.451724
H	11.837457	-0.552501	-2.936581
H	11.704887	4.869147	0.056122
H	11.290463	3.006523	-3.156409
H	11.496895	5.060420	-1.704876

H	11.330109	-4.271311	-3.170466
H	10.940944	-2.245049	1.387996
H	11.082599	-1.854662	-3.891682
H	10.869215	1.772149	1.964557
H	10.440557	1.860986	-2.085433
H	10.230628	4.262675	-0.743430
H	10.121815	-3.832235	-1.944152
H	10.246107	-1.167285	-2.474053
H	9.269832	2.387558	-0.616562
H	9.388496	-2.335233	2.644400
H	9.257392	1.705544	3.130473
H	9.191155	-2.196158	-1.164427
H	8.166101	-3.609559	2.402933
H	7.545924	1.249652	3.153063
H	8.006613	2.973442	3.097166
H	7.671814	-1.951632	2.844674
H	6.981833	2.289161	-0.537538
H	6.920720	-2.236745	-0.944203
H	5.702656	-2.412471	1.911735
H	5.617529	1.986157	2.239372
H	4.481333	2.232683	-0.651679
H	4.431982	-2.272897	-0.927496
H	3.749755	1.888150	3.073205
H	3.868812	-2.295110	2.842029
H	2.596965	3.200631	2.719202
H	2.723107	-3.597678	2.426135
H	2.037275	1.509065	2.831458
H	2.140914	-1.933467	2.710986
H	2.150845	2.183494	-0.968565
H	2.096511	-2.284623	-1.134334
H	0.309608	2.117893	1.546228
H	0.357086	-2.365831	1.452142
H	-0.306279	2.173833	-1.501966
H	-0.371604	-2.310678	-1.568866
H	-2.143469	2.154284	1.016620
H	-2.113244	-2.317216	1.017377
H	-2.039275	1.630458	-2.805114
H	-2.153918	-1.812814	-2.813837
H	-2.615510	3.311464	-2.630221
H	-2.713548	-3.493379	-2.588958
H	-3.756393	2.000262	-3.026553
H	-3.876617	-2.193813	-2.962103
H	-4.472656	2.214582	0.712317
H	-4.448411	-2.300927	0.804148
H	-5.712370	-2.314883	-2.041861
H	-5.624176	2.084579	-2.180408
H	-6.967498	2.273414	0.616981
H	-6.935933	-2.274338	0.816840
H	-7.557027	1.417580	-3.113052
H	-8.186112	-3.474626	-2.601394
H	-7.681961	-1.797860	-2.952560
H	-8.057077	3.125617	-2.970548
H	-9.248480	2.358395	0.726003
H	-9.400901	-2.181542	-2.772811
H	-9.277765	1.830262	-3.048080
H	-9.204316	-2.256685	1.036965
H	-10.375091	1.739849	2.181350
H	-10.194375	4.212761	0.982367
H	-10.139411	-3.955755	1.702587

H	-10.254994	-1.316534	2.406096
H	-10.957313	-2.157880	-1.512498
H	-11.211987	2.813445	3.333540
H	-10.887042	1.876564	-1.858263
H	-11.443673	4.949613	2.012132
H	-11.348118	-4.472343	2.898521
H	-11.070412	-2.114897	3.777235
H	-11.653187	-4.724278	1.158996
H	-11.989791	1.317988	2.757709
H	-11.682694	4.864427	0.246902
H	-11.843789	-0.749298	2.933402
H	-12.636647	-1.746284	-2.413959
H	-12.517822	1.274580	-2.727633
H	-13.442315	3.637967	2.802124
H	-13.762801	-3.052080	-1.961841
H	-13.299643	-3.009254	3.508521
H	-13.819961	4.187806	1.162982
H	-13.720577	2.581967	-2.585584
H	-13.751304	-3.868263	2.029089
H	-14.093887	1.258537	2.053631
H	-14.335937	-1.375876	-2.073584
H	-14.212339	0.888527	-2.381138
H	-14.000308	-0.820220	2.343695
H	-15.099486	2.745511	-0.440048
H	-15.166211	1.008329	-0.166621
H	-15.104336	-2.754286	0.227365
H	-15.160938	-0.995981	0.167695
H	-15.434597	2.413720	1.955198
H	-15.334502	-1.971443	2.524602

Supplementary Methods

General Consideration

All manipulations were conducted under a nitrogen atmosphere using standard Schlenk or drybox technique. ^{1}H and $^{13}C\{^{1}H\}$ NMR spectra were recorded on 400 MHz (JEOL JNM-ECS400) or 600 MHz (Varian Unity-Inova 600, JEOL JNM-ECA600) instruments. The chemical shifts were referenced to the residual resonances of deuterated solvents. Elemental analyses were performed at the Instrument Center of Institute for Molecular Science and X-ray crystal data were collected by Rigaku RAXIS-RAPID Imaging Plate diffractometer or Rigaku Saturn70 diffractometer at Institute for Molecular Science. ESI mass spectra were recorded on Bruker micrOTOF ESI-TOF. Unless specified all reagents were purchased from commercial suppliers and used without purification. All solvents were purified according to the standard procedures. *β*-Carotene was purified by recrystallization from benzene/MeOH. $[Pd_2(CH_3CN)_6][BF_4]_2$[1], $Pd_2(dba)_3{\cdot}(C_6H_6)$[2], $Pt_2(dba)_3{\cdot}(CHCl_3)$[3,4], sodium tetrakis[(3,5-trifluoro-methyl)phenyl]borate ($NaB(Ar^F)_4$)[5], and $[Pd_2$(1,4-diphenyl-1,3-butadiene)$_2][B(Ar^F)_4]_2$[6] were prepared according to the literature.

Synthesis of $[Pd_{10}$(*β*-carotene)$_2][B(Ar^F)_4]_2$ (1-meso) and (1-rac).

(1) A synthetic procedure under heating conditions: To a suspension of *β*-carotene (679 mg, 1.27 mmol) in $ClCH_2CH_2Cl$ (200 mL) were added $Pd_2(dba)_3{\cdot}(C_6H_6)$ (1.89 g, 1.90 mmol) and $[Pd_2(CH_3CN)_6][BF_4]_2$ (200 mg, 0.316 mmol) at room temperature. The reaction mixture was stirred under nitrogen atmosphere at 60 ºC for 1 day. The reaction mixture was filtered, and the filtrate was dried in vacuo. The obtained brown powder and $NaB(Ar^F)_4$ (560 mg, 0.632 mmol) was added to CH_2Cl_2, and the mixture was stirred for 5 min at room temperature. Et_2O was added to the solution and the mixture was filtered. The filtrate was dried in vacuo to yield a red powder. After washing with CH_3CN, $[Pd_{10}$(*β*-carotene)$_2][B(Ar^F)_4]_2$ (**1-meso**) was isolated as an yellow powder (290 mg, 24%). $[Pd_{10}$(*β*-carotene)$_2][B(Ar^F)_4]_2$ (**1-rac**) was obtained by recrystallization from the CH_3CN solution (107 mg, 9%).

(2) A synthetic procedure with visible light irradiation: To a suspension of *β*-carotene (63.8 mg, 118 μmol) in CH_2Cl_2 (20 mL) were added $Pd_2(dba)_3{\cdot}(C_6H_6)$ (257 mg, 259 μmol) and $[Pd_2(CH_3CN)_6][BF_4]_2$ (30 mg, 47.0 μmol) at room temperature. The reaction mixture was irradiated with visible light (Xenon lamp, >385 nm) at 20 ºC for 9 days.

After purifications in the same manner as those under heating conditions, **1-meso** and **1-rac** were obtained in 16% and 8% yields, respectively.

For **1-meso**: ^{1}H NMR (400 MHz, CD_2Cl_2, 25 ºC): δ –0.27 (s, 12H, H16), –0.15 (s, 12H, H19), 0.33 (s, 12H, H20), 1.54 (s, 12H, H18), 1.64 (m, 4H, H2), 1.78 (m, 4H, H3), 1.99 (m, 4H, H2'), 2.08 (m, 4H, H3'), 2.10 (s, 12H, H17), 2.65 (d, *J* = 12 Hz, 4H, H7), 2.66 (d, *J* = 12 Hz, 4H, H12), 2.86 (dd, *J* = 3 Hz, *J* = 9 Hz, 4H, H14), 2.95 (d, *J* = 12 Hz, 4H, H10), 3.04–3.11 (m, 8H, H8, H15), 3.28–3.45 (m, 8H, H4, H4'), 3.47 (t, *J* = 12 Hz, 4H, H11), 7.50 (s, 8H, *p-B(Ar^F)$_4$*), 7.65 (s, 16H, *o-B(Ar^F)$_4$*). ^{13}C NMR (100 MHz, CD_2Cl_2, 25 ºC): δ 14.3 (C19), 15.0 (C20), 20.5 (C3), 23.7 (C18), 28.1 (C16), 31.8 (C17), 34.9 (C1), 36.8 (C4), 43.5 (C2), 74.1 (C7), 74.7 (C8), 80.7 (C11), 82.1 (C15), 83.0 (C12), 83.3 (C10), 84.5 (C14), 98.0 (C13), 103.3 (C9), 109.7 (C6), 110.1 (C5), 117.8 (*p-B(Ar^F)$_4$*), 125.4 (*CF$_3$-B(Ar^F)$_4$*), 129.2 (*m-B(Ar^F)$_4$*), 135.1 (*o-B(Ar^F)$_4$*), 162.1 (*ipso-B(Ar^F)$_4$*). MS (ESI) *m/z* calcd. for $[C_{80}H_{112}Pd_{10}]^{2+}$: 1068.9601, Found: 1068.9647. Anal. Calcd. For $C_{144}H_{136}B_2F_{48}Pd_{10}$: C, 44.76; H, 3.55, Found: C, 44.77; H, 3.73. A single crystal suitable for X-ray crystallographic analysis was grown from a CH_2Cl_2 / toluene solution.

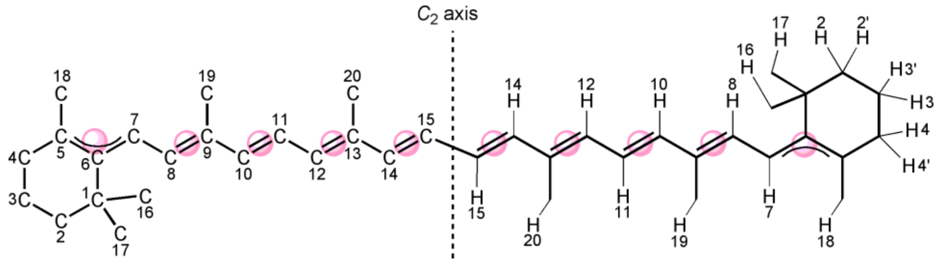

For **1-rac**: ^{1}H NMR (400 MHz, CD_2Cl_2, 25 ºC): δ –0.72 (s, 12H, H16), 0.20 (s, 12H, H19), 0.38 (s, 12H, H20), 1.40 (m, 4H, H2), 1.70 (m, 4H, H2′), 1.73 (s, 12H, H17), 1.80 (m, 4H, H3), 1.83 (s, 12H, H18), 2.08 (m, 4H, H3′), 2.77 (d, *J* = 12 Hz, 4H, H7), 2.80 (d, *J* = 12 Hz, 4H, H10), 2.85 (d, *J* = 12 Hz, 4H, H8), 3.03 (d, *J* = 12 Hz, 4H, H12), 3.07 (dd, *J* = 3 Hz, *J* = 9 Hz, 4H, H14), 3.33 (m, 4H, H4), 3.34 (t, *J* = 12 Hz, 4H, H11), 3.46 (dd, *J* = 3 Hz, *J* = 9 Hz, 4H, H15), 3.52 (m, 4H, H4'), 7.30 (s, 8H, *p-B(Ar^F)$_4$*), 7.65 (s, 16H, *o-B(Ar^F)$_4$*). ^{13}C NMR (100 MHz, CD_2Cl_2, 25 ºC): δ 13.7 (C19), 13.8 (C20), 20.0 (C3), 24.4 (C18), 29.4 (C16), 29.7 (C17), 34.4 (C1), 36.5 (C4), 43.1 (C2), 69.3 (C7), 76.5 (C11), 79.5 (C8), 84.0 (C15), 87.5 (C10), 88.1 (C12), 89.3 (C14), 95.0 (C9), 96.1 (C13), 99.4 (C5), 108.1 (C6), 117.8 (*p-B(Ar^F)$_4$*), 125.4 (*CF$_3$-B(Ar^F)$_4$*), 129.2 (*m-B(Ar^F)$_4$*), 135.1 (*o-B(Ar^F)$_4$*), 162.1 (*ipso-B(Ar^F)$_4$*). MS (ESI) *m/z* calcd. for $[C_{80}H_{112}Pd_{10}]^{2+}$: 1068.9601, Found: 1068.9432. Anal. Calcd. For $C_{144}H_{136}B_2F_{48}Pd_{10}\cdot C_6H_{14}$: C, 45.60; H,

3.83, Found: C, 45.48; H, 3.99. A single crystal suitable for X-ray crystallographic analysis was grown from a diethylether / hexane solution.

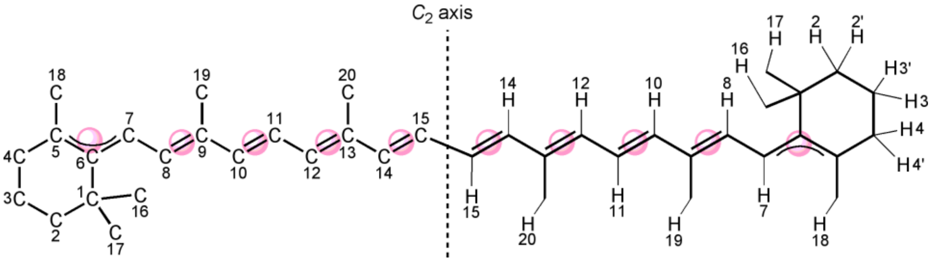

Synthesis of $[Pd_5(\beta\text{-carotene})_2][B(Ar^F)_4]_2$ (2-meso). CO gas (1 atm) was bubbled in a CH_2Cl_2 solution (220 mL) of $[Pd_{10}(\beta\text{-carotene})_2][B(Ar^F)_4]_2$ (**1-meso**) (340 mg, 88.0 μmol) at 5 ºC for 24 h. The reaction mixture was filtered and the filtrate was dried in vacuo to give a dark brown powder. After extraction with CH_3CN, the volatiles were removed in vacuo to give $[Pd_5(\beta\text{-carotene})_2][B(Ar^F)_4]_2$ (**2-meso**) as a dark brown powder (218 mg, 74%). ^{1}H NMR (400 MHz, CD_2Cl_2, 25 ºC): δ –0.29 (s, 6H, H31), 0.05 (s, 6H, H34), 0.90 (s, 6H, H35), 1.05 (s, 6H, H39), 1.06 (s, 6H, H40), 1.47 (m, 4H, H29), 1.60 (s, 6H, H33), 1.60–1.68 (m, 8H, H2, H3, H28), 1.74 (s, 6H, H38), 1.92–2.08 (m, 6H, H2', H27), 1.94 (s, 6H, H36), 1.98 (s, 6H, H32), 2.02 (d, J = 12 Hz, 2H, H12), 2.06 (s, 6H, H37), 2.12 (m, 2H, H3'), 2.39 (d, J = 12 Hz, 2H, H14), 2.52 (d, J = 12 Hz, 2H, H10), 2.92 (t, J = 12 Hz, 2H, H8), 2.96 (t, J = 12 Hz, 2H, H7), 3.3 (m, 4H, H4), 3.67 (t, J = 12 Hz, 2H, H11), 5.13 (t, J = 12 Hz, 2H, H15), 5.85 (t, J = 12 Hz, 2H, H16), 6.10 (d, J = 12 Hz, 2H, H17), 6.15 (d, J = 16 Hz, 2H, H23), 6.22 (d, J = 12 Hz, 2H, H21), 6.26 (d, J = 16 Hz, 2H, H19), 6.29 (d, J = 16 Hz, 2H, H24), 6.91 (dd, J = 12 Hz, J = 16 Hz, 2H, H20), 7.49 (s, 8H, *p-B(Ar^F)$_4$*), 7.64 (s, 16H, *o-B(Ar^F)$_4$*). ^{13}C NMR (100 MHz, CD_2Cl_2, 25 ºC): δ 13.0 (C37), 13.3 (C36), 14.0 (C35), 14.2 (C34), 19.6 (C28), 19.9 (C3), 22.1 (C38), 24.0 (C33), 28.0 (C31), 29.2 (C39), 29.4 (C40), 31.6 (C32), 33.7 (C27), 34.6 (C30), 35.0 (C1), 36.8 (C4), 40.1 (C29), 42.7 (C2), 76.8 (C8), 78.9 (C7), 79.7 (C11), 89.2 (C14), 89.4 (C13), 89.6 (C10), 91.8 (C12), 92.5 (C15), 103.5 (C9), 111.8 (C16), 112.3 (C6), 117.8 (*p-B(Ar^F)$_4$*), 123.4 (C5), 125.5 (*CF$_3$-B(Ar^F)$_4$*), 126.7 (C17), 129.2 (*m-B(Ar^F)$_4$*), 129.3 (C26), 129.9 (C24), 130.4 (C20), 130.6 (C21), 135.1 (*o-B(Ar^F)$_4$*), 135.7 (C19), 137.6 (C23), 138.0 (C25), 140.3 (C22), 143.9 (C18), 162.1 (*ipso-B(Ar^F)$_4$*). MS (ESI) *m/z* calcd. for $[C_{80}H_{112}Pd_5]^{2+}$: 802.6996, Found: 802.7014. Anal. Calcd. For $C_{144}H_{136}B_2F_{48}Pd_5 \cdot C_6H_6$: C, 52.83; H, 4.20, Found: C, 52.65; H, 4.35. A single crystal suitable for X-ray crystallographic analysis was grown from a diethylether / benzene solution.

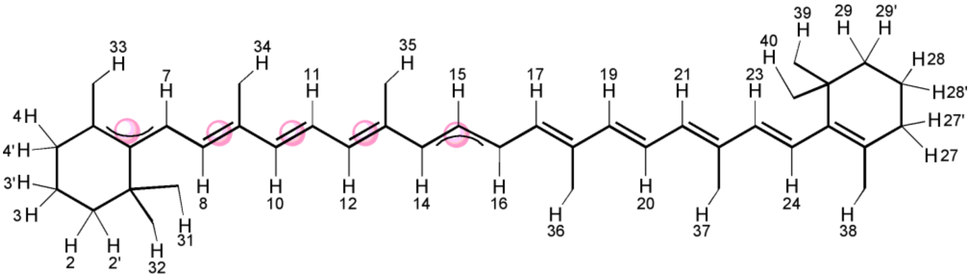

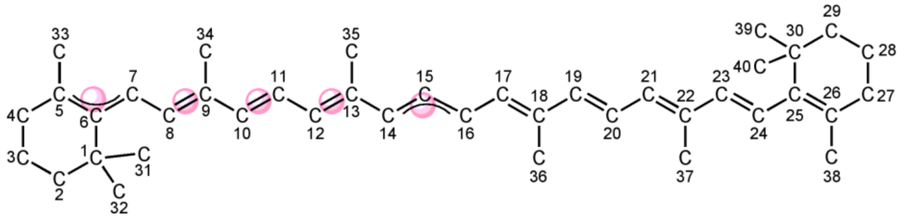

Synthesis of [Pd_7(*β*-carotene)$_2$][$B(Ar^F)_4$]$_2$ (3-meso). CO gas (1 atm) was bubbled in a CH_2Cl_2 solution (100 mL) of [Pd_{10}(*β*-carotene)$_2$][$B(Ar^F)_4$]$_2$ (**1-meso**) (107 mg, 27.8 μmol) at 30 ºC for 3 h in the dark. The reaction mixture was filtered and the filtrate was dried in vacuo to give a dark red powder. The resultant powder was washed with CH_3CN, and dried in vacuo. Et_2O was added and the mixture was filtered. The filtrate was dried in vacuo to give [Pd_7(*β*-carotene)$_2$][$B(Ar^F)_4$]$_2$ (**3-meso**) as a red powder (20.0 mg, 20%). ^{1}H NMR (400 MHz, CD_2Cl_2, 25 ºC): δ –0.27 (s, 6H, H31), –0.04 (s, 6H, H34), 0.07 (s, 6H, H36), 0.67 (s, 6H, H35), 1.12 (s, 6H, H39), 1.13 (s, 6H, H40), 1.51 (m, 4H, H29), 1.59 (s, 6H, H33), 1.61–1.70 (m, 6H, H2, H28), 1.79 (s, 6H, H38), 1.97 (m, 2H, H2'), 2.06 (s, 6H, H32), 2.06–2.15 (m, 8H, H3, H27), 2.23 (d, J = 12 Hz, 2H, H14), 2.33 (d, J = 12 Hz, 2H, H12), 2.34 (s, 6H, H37), 2.40 (t, J = 12 Hz, 2H, H16), 2.79 (d, J = 12 Hz, 2H, H10), 2.85 (d, J = 12 Hz, 2H, H7), 3.03 (d, J = 12 Hz, 2H, H8), 3.27 (d, J = 12 Hz, 2H, H17), 3.34 (m, 4H, H4), 3.56 (t, J = 12 Hz, 2H, H15), 3.65 (d, J = 12 Hz, 2H, H11), 4.50 (d, J = 12 Hz, 2H, H19), 6.08 (d, J = 12 Hz, 2H, H21), 6.28 (d, J = 16 Hz, 2H, H23), 6.44 (d, J = 16 Hz, 2H, H24), 6.46 (t, J = 12 Hz, 2H, H20), 7.48 (s, 8H, *p-B(Ar^F)$_4$*), 7.63 (s, 16H, *o-B(Ar^F)$_4$*). ^{13}C NMR (100 MHz, CD_2Cl_2, 25 ºC): δ 13.6 (C37), 14.4 (C34), 14.6 (C35), 14.7 (C36), 19.6 (C28), 20.2 (C3), 22.2 (C38), 23.9 (C33), 28.0 (C31), 29.3 (C39), 29.5 (C40), 31.7 (C32), 34.0 (C27), 34.5 (C30), 34.9 (C1), 36.7 (C4), 40.2 (C29), 43.0 (C2), 75.5 (C7), 76.8 (C8), 79.0 (C15), 80.3 (C17), 80.6 (C11), 86.7 (C10), 87.9 (C12), 88.8 (C16), 89.2 (C14), 95.5 (C13), 96.1 (C19), 96.4 (C20), 100.9 (C18), 103.5 (C9), 111.2 (C6), 117.1 (C5), 117.8 (*p-B(Ar^F)$_4$*), 125.5 (*CF_3-B(Ar^F)$_4$*), 127.2 (C21), 129.2 (*m-B(Ar^F)$_4$*), 129.4 (C24), 131.5 (C26), 135.1 (*o-B(Ar^F)$_4$*), 136.6 (C23), 137.6 (C22), 140.4 (C25), 162.1 (*ipso-B(Ar^F)$_4$*).

MS (ESI) *m/z* calcd. for $[C_{80}H_{112}Pd_7]^{2+}$: 909.1038, Found: 909.1082. Anal. Calcd. For $C_{144}H_{136}B_2F_{48}Pd_7{\cdot}(C_6H_6)_2$: C, 50.62; H, 4.03, Found: C, 50.73; H, 4.06. A single crystal suitable for X-ray crystallographic analysis was grown from a diethylether / benzene solution.

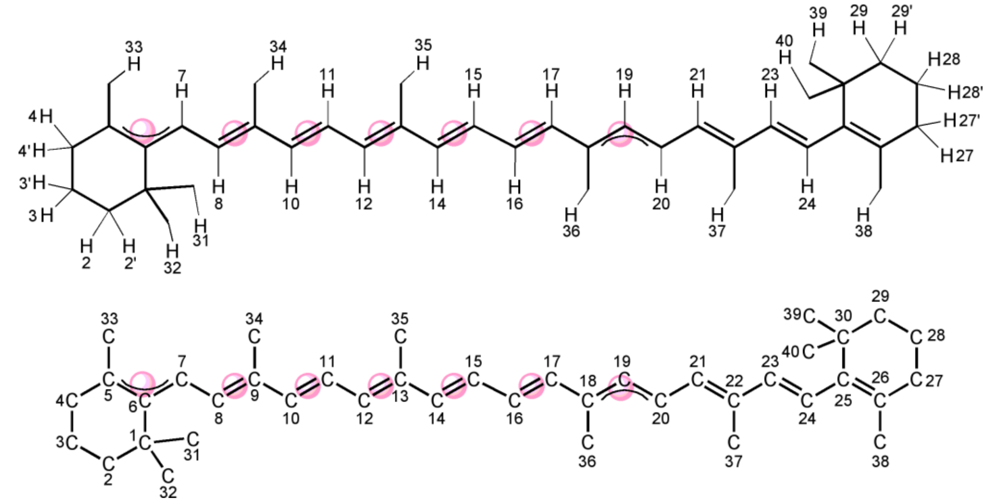

Synthesis of $[Pd_5Pt_3(\beta\text{-carotene})_2][B(Ar^F)_4]_2$ (4-meso). Ethylene gas (1 atm) was bubbled in a CH_2Cl_2 solution (30 mL) of $[Pd_5(\beta\text{-carotene})_2][B(Ar^F)_4]_2$ (**2-meso**) (54.0 mg, 16.2 μmol) and $Pt_2(dba)_3{\cdot}(CHCl_3)$ (100 mg, 82.5 μmol) at r.t. for 5 min. After the solution was stirred at 30 ºC for 1 day, the color turned dark brown. The reaction mixture was dried in vacuo to give a dark brown powder. After extraction with Et_2O and washing with C_6H_6, $[Pd_5Pt_3(\beta\text{-carotene})_2][B(Ar^F)_4]_2$ (**4-meso**) was isolated as a yellow solid (37.6 mg, 59%). ^{1}H NMR (400 MHz, CD_2Cl_2, 25 ºC): δ –0.28 (s, 6H, H31), –0.11 (s, 6H, H34), 0.50 (s, 12H, H35, H36), 1.17 (s, 6H, H39), 1.21 (s, 6H, H40), 1.56 (s, 6H, H33), 1.56–1.59 (m, 4H, H29, H29'), 1.60–1.72 (m, 8H, H2, H3, H28, H28'), 1.84 (s, 6H, H38), 1.98 (m, 2H, H2'), 2.01 (s, 6H, H37), 2.07 (s, 6H, H32), 2.09 (m, 2H, H3'), 2.13–2.18 (m, 4H, H27, H27'), 2.34 (d, *J* = 12 Hz, 2H, H12), 2.36 (d, *J* = 12 Hz, 2H, H14), 2.43 (t, *J* = 12 Hz, 2H, H16), 2.71 (d, *J* = 12 Hz, 2H, H19), 2.76 (d, *J* = 14 Hz, 2H, H7), 2.81 (d, *J* = 12 Hz, 2H, H10), 3.00 (t, *J* = 11 Hz, 2H, H20), 3.04 (d, *J* = 12 Hz, 2H, H8), 3.11 (d, *J* = 12 Hz, 2H, H17), 3.3 (m, 4H, H4), 3.40 (m, 2H, H15), 3.48 (t, *J* = 12 Hz, 2H, H11), 3.79 (d, *J* = 11 Hz, 2H, H21), 6.47 (d, *J* = 16 Hz, 2H, H23), 6.68 (d, *J* = 16 Hz, 2H, H24), 7.49 (s, 8H, *p-B(Ar^F)$_4$*), 7.65 (s, 16H, *o-B(Ar^F)$_4$*). ^{13}C NMR (100 MHz, CD_2Cl_2, 25 ºC): δ 14.3 (C34), 14.6 (C35), 15.5 (C36), 17.5 (C37), 19.7 (C28), 20.3 (C3), 22.2 (C38), 23.8 (C33), 28.0 (C31), 29.2 (C39), 29.7 (C40), 31.7 (C32), 33.9 (C27), 34.7 (C30), 34.9 (C1), 36.8 (C4), 40.1 (C29), 43.2 (C2), 64.4 (C19), 67.3 (C17), 74.9

(C7), 75.1 (C16), 75.9 (C8), 78.1 (C20), 78.7 (C15), 79.9 (C21), 80.6 (C11), 85.2 (C10), 85.7 (C12), 87.6 (C14), 96.9 (C13), 98.4 (C22), 98.6 (C18), 103.5 (C9), 110.6 (C6), 114.4 (C5), 117.8 (*p-B(Ar^F)$_4$*), 125.5 (*CF$_3$-B(Ar^F)$_4$*), 129.1 (C24), 129.2 (*m-B(Ar^F)$_4$*), 132.3 (C26), 135.1 (*o-B(Ar^F)$_4$*), 136.0 (C23), 136.5 (C25), 162.1 (*ipso-B(Ar^F)$_4$*). MS (ESI) *m/z* calcd. for $[C_{80}H_{112}Pd_5Pt_3]^{2+}$: 1095.1460, Found: 1095.1291. Anal. Calcd. For $C_{144}H_{136}B_2F_{48}Pd_5Pt_3\cdot(C_6H_6)_2$: C, 45.99; H, 3.66, Found: C, 46.10; H, 3.86. A single crystal suitable for X-ray crystallographic analysis was grown from a dichloromethane / benzene solution.

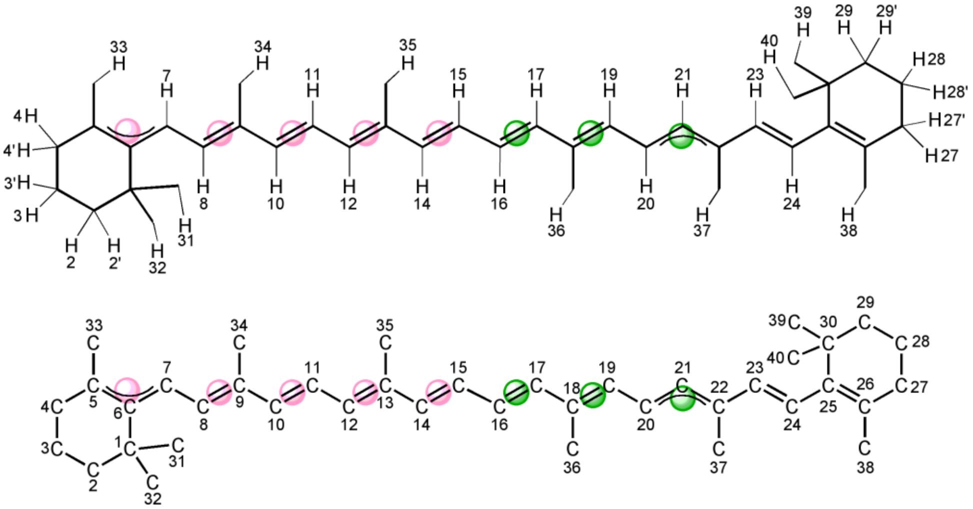

Synthesis of [$Pd_5Pt_3Pd_2$(*β*-carotene)$_2$][B(Ar^F)$_4$]$_2$ (5-meso). To a solution of [Pd_5Pt_3(*β*-carotene)$_2$][B(Ar^F)$_4$]$_2$ (**4-meso**) (58.0 mg, 14.8 μmol) in $ClCH_2CH_2Cl$ (50 mL) was added $Pd_2(dba)_3\cdot(C_6H_6)$ (200 mg, 201 μmol), and the reaction mixture was stirred under nitrogen atmosphere at 70 ºC for 12 h. The mixture was filtered, and the filtrate was dried in vacuo. After reprecipitation with CH_2Cl_2 / hexane, the yellow powder was obtained. After drying in vacuo, the product was analyzed by NMR, showing that [$Pd_5Pt_3Pd_2$(*β*-carotene)$_2$][B(Ar^F)$_4$]$_2$ (**5-meso**) was formed as a major product with an unidentified minor product (major:minor = 8:2) (a mixture of two products: 43 mg). For **5-meso**: ^{1}H NMR (600 MHz, CD_2Cl_2, 25 ºC): δ –0.27 (s, 6H, H31 or 40), –0.22 (s, 6H, H31 or 40), –0.17 (s, 6H, H34), 0.11 (s, 6H, H37), 0.31 (s, 6H, H35), 1.01 (s, 6H, H36), 1.48 (s, 6H, H33 or 38), 1.54 (s, 6H, H33 or 38), 1.63 (m, 4H, H2, 29), 1.78 (m, 4H, H3, 28), 1.98 (m, 4H, H2', 29'), 2.05 (s, 6H, H32 or 39), 2.08 (m, 4H, H3', 28'), 2.09 (s, 6H, H32 or 39), 2.52 (d, *J* = 12 Hz, 2H, H12), 2.56 (d, *J* = 12 Hz, 2H, H24), 2.60 (t, *J* = 12 Hz, 2H, H16), 2.62 (d, *J* = 12 Hz, 2H, H14), 2.66 (d, *J* = 12 Hz, 2H, H7), 2.74 (d, *J* = 12 Hz, 2H, H21), 2.90 (d, *J* = 12 Hz, 4H, H10, 19), 2.91 (d, *J* = 12

Hz, 2H, H17), 2.95 (d, $J = 12$ Hz, 2H, H23), 3.07 (d, $J = 12$ Hz, 2H, H8), 3.19 (t, $J = 12$ Hz, 2H, H15), 3.30 (m, 8H, H4, 4', 27, 27'), 3.40 (t, $J = 12$ Hz, 2H, H11), 3.42 (t, $J = 12$ Hz, 2H, H20), 7.51 (s, 8H, *p-BAr$^F_4$*), 7.66 (s, 16H, *o-BAr$^F_4$*). ^{13}C NMR (150 MHz, CD_2Cl_2, 25 ºC): δ 14.1 (C37), 14.3 (C34), 14.8 (C35), 15.8 (C36), 20.4 (C3 or 28), 20.5 (C3 or 28), 23.7 (C33, 38), 28.1 (C31 or 40), 28.2 (C31 or 40), 31.8 (C32 or 39), 31.9 (C32 or 39), 34.9 (C1, C30), 36.8 (C4 or 27), 36.9 (C4 or 27), 43.4 (C2 or 29), 43.5 (C2 or 29), 70.0 (C19), 71.1 (C16), 71.2 (C17), 71.3 (C20), 71.8 (C21), 73.6 (C23), 74.1 (C7), 74.6 (C24), 74.8 (C8), 80.5 (C11), 81.8 (C15), 83.1 (C12), 83.4 (C10), 84.6 (C14), 89.9 (C18), 98.2 (C13), 103.5 (C9), 105.3 (C22), 109.8 (C6), 110.4 (C5, C25), 111.4 (C26), 117.9 (*p-BAr$^F_4$*), 125.0 (*CF$_3$-BAr$^F_4$*), 129.2 (*m-BAr$^F_4$*), 135.2 (*o-BAr$^F_4$*), 162.1 (*ipso-BAr$^F_4$*). MS (ESI) *m/z* calcd. for $[C_{80}H_{112}Pd_7Pt_3]^{2+}$: 1201.5503, Found: 1201.5553. Anal. Calcd. For $C_{144}H_{136}B_2F_{48}Pd_7Pt_3{\cdot}(C_6H_6)$: C, 42.81; H, 3.40, Found: C, 42.87; H, 3.46. The sample suitable for elemental analysis was obtained from a dichloromethane / benzene solution. The minor product is probably an isomer of **5-meso** having a different metal arrangement.

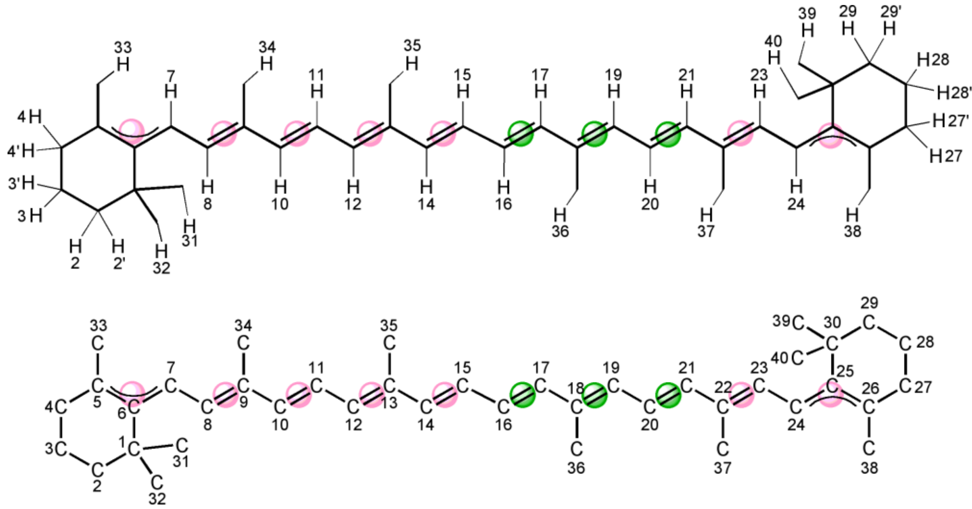

Synthesis of $[Pd_2(1,10\text{-diphenylpentaene})_2][B(Ar^F)_4]_2$. To a suspension of 1,10-diphenyl-1,3,5,7,9-decapentaene (80.0 mg, 0.28 mmol) in CH_2Cl_2 (30 mL) was added $[Pd_2(1,4\text{-diphenyl-1,3-butadiene})][B(Ar^F)_4]_2$ (299.6 mg, 0.13 mmol). The mixture was stirred for 1 h at room temperature. The reaction mixture was filtered and poured into hexane to give deep-green precipitation. $[Pd_2(1,10\text{-diphenylpentaene})_2][B(Ar^F)_4]_2$ was isolated as a mixture of four isomers by recrystallization (262.1 mg, 82%, isomer ratio 57:27:10:6). ^{1}H NMR (600 MHz, CD_2Cl_2, 25 ºC) of vinyl protons in **A** (57% of products): δ 3.30 (dd, $J = 12$ Hz, $J = 12$ Hz, 1H, H3'), 3.38 (dd, $J = 12$ Hz, $J = 12$ Hz,

1H, H5), 3.54 (dd, *J* = 12 Hz, *J* = 12 Hz, 1H, H6), 3.75 (dd, *J* = 11 Hz, *J* = 12 Hz, 1H, H4'), 4.29 (dd, *J* = 11 Hz, *J* = 13Hz, 1H, H5'), 4.33 (dd, *J* = 11 Hz, *J* = 12 Hz, 1H, H7), 4.48 (dd, *J* = 12 Hz, *J* = 13 Hz, 1H, H4), 5.07 (dd, *J* = 12 Hz, *J* = 14 Hz, 1H, H2'), 5.52 (dd, *J* = 12 Hz, *J* = 13 Hz, 1H, H6'), 5.77 (dd, *J* = 11 Hz, *J* = 13 Hz, 1H, H3), 5.90 (dd, *J* = 11 Hz, *J* = 15 Hz, 1H, H9), 6.05 (dd, *J* = 12 Hz, *J* = 14 Hz, 1H, H7'), 6.08 (dd, *J* = 11 Hz, *J* = 15 Hz, 1H, H2), 6.14 (d, *J* = 14 Hz, 1H, H1'), 6.21 (dd, *J* = 11 Hz, *J* = 15 Hz, 1H, H9'), 6.40 (dd, *J* = 11 Hz, *J* = 11 Hz, 1H, H8), 6.47 (dd, *J* = 11 Hz, *J* = 14 Hz, 1H, H8'), 6.50 (d, *J* = 15 Hz, 1H, H1), 6.69 (d, *J* = 15 Hz, 1H, H10'), 6.71 (dd, *J* = 15 Hz, 1H, H10). **B** or **C** (27% of products): δ 3.34 (dd, *J* = 11 Hz, *J* = 11 Hz, 2H, H3), 3.69 (dd, *J* = 11 Hz, *J* = 11 Hz, 2H, H4), 4.32 (dd, *J* = 11 Hz , *J* = 13 Hz, 2H, H5), 4.98 (dd, *J* = 11 Hz, *J* = 14 Hz, 2H, H2), 5.46 (dd, *J* = 12 Hz, *J* = 14 Hz, 2H, H7), 5.76 (dd, *J* = 12 Hz, 13 Hz, 2H, H6), 6.21 (dd, *J* = 11 Hz, *J* = 14 Hz, 2H, H8), 6.42 (d, *J* = 14 Hz, 2H, H1), 6.61 (dd, *J* = 11 Hz, *J* = 15 Hz, 2H, H9), 6.80 (dd, *J* = 15 Hz, 2H, H10). **B** or **C** (10% of products): δ 3.30 (dd, *J* = 11 Hz, *J* = 11 Hz, 2H, H3), 3.78 (dd, *J* = 11 Hz, *J* = 11 Hz, 2H, H4), 4.23 (dd, *J* = 11 Hz, *J* = 13 Hz, 2H, H5), 5.03 (dd, *J* = 11 Hz, *J* = 14 Hz, 2H, H2), ca. 5.46 (2H, H7), 6.13 (d, *J* = 14 Hz, 2H, H1), 6.31 (dd, *J* = 11 Hz, *J* = 15 Hz, 2H, H9), ca. 6.51 (2H, H6), 6.56 (dd, *J* = 11 Hz, *J* = 14 Hz, 2H, H8), 7.01 (dd, *J* = 15 Hz, 2H, H10). **D** (6% of products): δ 3.48 (m, 4H, H5), 4.49 (m, 4H, H4), 6.01 (dd, *J* = 11 Hz, *J* = 13 Hz, 4H, H3), 6.18 (dd, *J* = 11 Hz, *J* = 15 Hz, 4H, H2), 6.69 (d, *J* = 15.6 Hz, 4H, H1). Anal. Calcd. For $C_{108}H_{64}B_2F_{48}Pd_2$: C, 51.72; H, 2.57, Found: C, 51.59; H, 2.58.

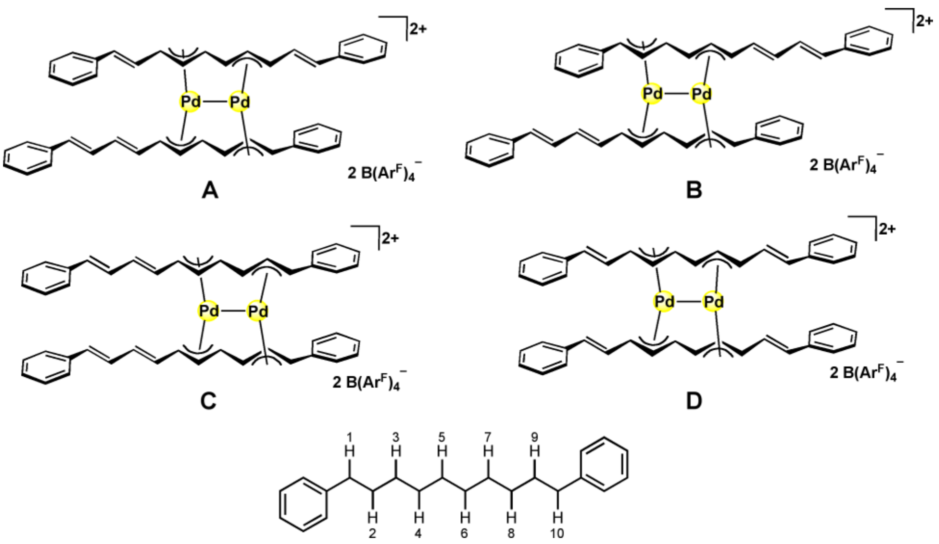

Crystal Data

Metrical parameters for the crystal structures of **1-meso**, **1-rac, 2-meso**, **3-meso**, and **4-meso** are available free of charge from the Cambridge Crystallographic Data Centre under reference numbers CCDC-1001723 (**1-meso**), 1001724 (**1-rac**), 1001725 (**2-meso**), 1001726 (**3-meso**), and 1001727 (**4-meso**), respectively.

Crystal data for **1-meso**. $C_{144}H_{136}B_2F_{48}Pd_{10}\cdot(C_7H_8)_2$, M = 4048.48, space group $P2_1/n$ (no. 14), a = 10.5110(9), b = 20.522(2), c = 35.439(3) Å, β = 94.2797(13)°, V = 7623.1(11) Å^3, Z = 2, F(000) = 4004, Dc = 1.764 g/cm^3, μ(MoKa) = 12.60 cm^{-1}, T = 133 K, 988 variables refined with 14750 reflections with $I > 2\sigma(I)$ to $R1$ = 0.0409. Solvent toluene molecules were disordered. They were refined isotropically on two positions (61:39). Restraints instructions (DFIX, FLAT) were applied in the treatment of disorder.

Crystal data for **1-rac**. $C_{144}H_{136}B_2F_{48}Pd_{10}\cdot(C_4H_{10}O_2)_2$, M = 4122.66, space group $P\bar{1}$ (no. 2), a = 19.438(1), b = 21.753(2), c = 22.055(2) Å, α = 86.871(1)°, β = 84.951(1)°, γ = 66.930(1)°, V = 8544.5(8) Å^3, Z = 2, F(000) = 3952, Dc = 1.556 g/cm^3, μ(MoKa) = 11.23 cm^{-1}, T = 123 K, 1825 variables refined with 30707 reflections with $I > 2\sigma(I)$ to $R1$ = 0.0878. This structure was treated with SQUEEZE protocol inside PLATON because the crystal lattice contains large voids filled with scattered electron density. 4 $CH_3CH_2OCH_2CH_3$ molecules per unit cell that were omitted from the model were included in the formula for calculation of the intensive properties. Restraints instructions (SAME, SIMU, DELU) were applied to the counter anions.

Crystal data for **2-meso**. $C_{144}H_{136}B_2F_{48}Pd_5\cdot(C_6H_6)_3$, M = 3566.54, space group $P\bar{1}$ (no. 2), a = 17.1352(6), b = 20.7763(7), c = 23.2367(9) Å, α = 104.5810(8)°, β = 90.8059(8)°, γ = 105.7605(7)°, V = 7674.8(5) Å^3, Z = 2, F(000) = 3596, Dc = 1.543 g/cm^3, μ(MoKa) = 6.84 cm^{-1}, T = 123 K, 1950 variables refined with 29864 reflections with $I > 2\sigma(I)$ to $R1$ = 0.0792. A CF_3 group in a counter anion was refined with restraints instructions (SIMU, DELU). For a comment on an alert level B in a checkcif: [PLAT201_ALERT_2_B], two disordered CF_3 groups in counter anions were refined isotropically on two positions, respectively.

Crystal data for **3-meso**. $C_{144}H_{136}B_2F_{48}Pd_7{\cdot}(C_6H_6)_3$, M = 3779.34, space group $P\bar{1}$ (no. 2), a = 17.134(2), b = 20.748(2), c = 23.226(2) Å, α = 104.413(2)°, β = 90.673(2)°, γ = 105.167(2)°, V = 7693(2) Å^3, Z = 2, F(000) = 3780, Dc = 1.632 g/cm^3, μ(MoKa) = 9.10 cm^{-1}, T = 123 K, 1968 variables refined with 28724 reflections with $I > 2\sigma(I)$ to $R1$ = 0.0853. For comments on alert level B in a checkcif: [DIFMX01_ALERT_2_B] and [PLAT097_ALERT_2_B], the large residual electron density was located near Pd7, C20, C21, C50 and C51. This is possibly derived from contamination of a small amount of a Pd_8 complex; [PLAT201_ALERT_2_B], two disordered CF_3 groups in counter anions were refined isotropically on two positions, respectively.

Crystal data for **4-meso**. $C_{144}H_{136}B_2F_{48}Pd_5Pt_3{\cdot}(C_6H_6)_3$, M = 4151.81, space group $P\bar{1}$ (no. 2), a = 17.031(2), b = 20.825(2), c = 23.393(2) Å, α = 75.288(2)°, β = 89.972(2)°, γ = 74.276(2)°, V = 7704(1) Å^3, Z = 2, F(000) = 4064, Dc = 1.790 g/cm^3, μ(MoKa) = 33.78 cm^{-1}, T = 123 K, 1926 variables refined with 28612 reflections with $I > 2\sigma(I)$ to $R1$ = 0.0976. The terminal group of the β-carotene ligand was refined with restraints instructions (SIMU, DELU). Several carbon atoms and fluorine atoms of counter anions were refined with restraints instructions (SIMU, DELU). Three benzene molecules were refined with restraints instructions (SIMU, DELU). For comments on alert level B in a checkcif: [PLAT201_ALERT_2_B], the disordered CF_3 group in the counter anion was refined isotropically on two positions; [PLAT342_ALERT_3_B], this alert is caused by the disorder in the structure.

NOTE: All cif files included an alert level A about the author's number: [PUBL024_ALERT_1_A]. For the roles of authors, the idea and plans of this research were made by T.Mu. Experiments and data analysis were performed by S.H., Y.T., M.Y., K.Y., K.M., K.T., T.Ma., S.K., and T.Mu. The theoretical calculations were performed by Y.K. and T.Y. The manuscript was co-written by T.Mu., Y.K., and T.Y. All authors discussed the results.

Computational Details

Quantum chemical investigation was performed on $[Pd_m(\beta\text{-carotene})_2]^{2+}$ for m = 5, 7, and 10 using density functional theory (DFT). All the DFT calculations were carried out with the ORCA software package (version 3.0.1)[7]. The details of computational settings are given as follows:

- The def2-TZVP (Pd atoms) and def2-SV(P) (C and H atoms) basis sets were employed for atomic orbital basis functions[8,9].
- The def2-TZVP/J (Pd atoms) and def2-SVP/J (C and H stoms) basis sets were used for the auxiliary basis functions in the resolution-of-identity (RI) approximation to electronic repulsion integrals (ERIs)[10].
- The effective core potential (ECP) approximation was used for Pd atoms with the parameter set [SD(28,MWB)][11].
- The total net charge was set to +2 throughout this DFT study; thus, the systems are hereafter denoted as $[Pd_m(\beta\text{-carotene})_2]^{2+}$. The spin multiplicity was specified to be singlet.
- Optimized geometries of $[Pd_m(\beta\text{-carotene})_2]^{2+}$ were obtained for the electronic ground states in the gas-phase using the hybrid B3LYP functional[12]. In all optimizations, semi-empirical corrections with zero damping to account for atom-pairwise (atom-triplewise) dispersion forces[13] were added to the DFT energies (`D3ZERO` option in ORCA 3.0.1).
- With the optimized geometries, vertical electronic excitations were calculated on the basis of time-dependent DFT (TDDFT) using the Coulomb-attenuated (or long-range-corrected) CAM-B3LYP functional[14].
- In order to accelerate ERI evaluation, the RIJCOSX[15] and RIJONX[16] approximations were employed in the B3LYP and CAM-B3LYP calculations, respectively.

The results obtained from the DFT and TDDFT calculations are summarized briefly as follows:

- Surface plots of HOMO−i and LUMO+i of $[Pd_m(\beta\text{-carotene})_2]^{2+}$ for i = 0,..,4 are shown along with their orbital energies in **Supplementary Figures 5** (m = 5), **6** (m = 7), and **7** (m = 10).
- **Supplementary Table 2** shows the energies and characters of the low-lying excited

states that were predicted to have appreciable oscillator strength.

- **Supplementary Figure 4** displays difference densities of the excited states relative to the ground state. They indicate how electron distribution is altered by the excitation.
- The Cartesian coordinates of the optimized molecular structures of [Pd_m(*β*-carotene)$_2$] for *m* = 5, 7, and 10 are provided in **Supplementary Tables 3-5**, respectively.

Supplementary References

1. Murahashi, T., Nagai, T., Okuno, T., Matsutani, T., Kurosawa, H. Synthesis and ligand substitution reactions of a homoleptic acetonitrile dipalladium(I) complex. *Chem. Commun.* 1689–1690 (2000).
2. Ukai, T., Kawazura, H., Ishii, Y., Bonnet, J. J., Ibers, J. A. A. Chemistry of dibenzylideneacetone-palladium(0) complexes: I. Nobel tris(dibenzylideneacetone)dipalladium(solvent) complexes and their reactions with quinones. *J. Organomet. Chem.* **65**, 253–266 (1974).
3. Moseley, K., Maitlis, P. M. Acetylenes and noble metal compounds. Part XI. Reactions of di-methyl acetylenedicarboxylate with dibenzylideneacetone–palladium and –platinum complexes: pallada- and platina-cyclopentadienes. *J. Chem. Soc., Dalton Trans*. 169–175 (1974).
4. Tanaka, H., Kawazura, H. NMR Studies on Zerovalent Metal π-Complexes of Dibenzylideneacetone. II. Structure and Pt–H Coupling in the Binuclear Platinum Complex. *Bull. Chem. Soc. Jpn.* **52**, 2815–2818 (1979).
5. Yakelis, N. A., Bergman, R. G. Safe Preparation and Purification of Sodium Tetrakis[(3,5-trifluoromethyl)phenyl]borate ($NaBArF_{24}$): Reliable and Sensitive Analysis of Water in Solutions of Fluorinated Tetraarylborates. *Organometallics*, **24**, 3579–2581 (2005).
6. Tatsumi, Y., Nagai, T., Nakashima, H., Murahashi, T., Kurosawa, H. Stepwise growth of polypalladium chains in 1,4-diphenyl-1,3-butadiene sandwich complexes. *Chem. Commun.* 1430–1431 (2004).
7. Neese, F. The ORCA program system. *WIREs Comp. Mol. Sci.* **2**, 73–78 (2012).

8. Schaefer, A., Horn H., and Ahlrichs, R. Fully optimized contracted Gaussian basis sets for atoms Li to Kr. *J. Chem. Phys.* **97**, 2571–2577 (1992).
9. Weigend, F., Ahlrichs, R. Balanced basis sets of split valence, triple zeta valence and quadruple zeta valence quality for H to Rn: Design and assessment of accuracy. *Phys. Chem. Chem. Phys.* **7**, 3297–3305 (2005).
10. Weigend F. Accurate Coulomb-fitting basis sets for H to Rn. *Phys. Chem. Chem. Phys.* **8**, 1057–1065 (2006).
11. Andrae, D., Haeussermann, U., Dolg, M., Stoll, H., Preuss, H. Energy-adjusted*ab initio* pseudopotentials for the second and third row transition elements. *Theor. Chim. Acta* **77**, 123–141 (1990).
12. Becke, A. D. Density- functional thermochemistry. III. The role of exact exchange. *J. Chem. Phys.* **98**, 5648–5652 (1993).
13. Grimme, S., Antony, J., Ehrlich, S., Krieg, H. A consistent and accurate *ab initio* parametrization of density functional dispersion correction (DFT-D) for the 94 elements H-Pu. *J. Chem. Phys.* **132**, 154104 (2010).
14. Yanai, T., Tew, D., Handy, N. A new hybrid exchange–correlation functional using the Coulomb-attenuating method (CAM-B3LYP). *Chem. Phys. Lett.* **393**, 51–57 (2004).
15. Neese, F., Wennmohs, F., Hansen, A., Becker, U. Efficient, approximate and parallel Hartree–Fock and hybrid DFT calculations. A 'chain-of-spheres' algorithm for the Hartree–Fock exchange. *Chem. Phys.* **356**, 98–109 (2009).
16. Kendall, R. A., Fruchtl, H. A. The impact of the resolution of the identity approximate integral method on modern ab initio algorithm development. *Theor. Chem. Acc.* **97**, 158–163 (1997).
